# CRISPR/Cas9 screen for genome‐wide interrogation of essential MYC‐bound E‐boxes in cancer cells

**DOI:** 10.1002/1878-0261.13493

**Published:** 2023-08-07

**Authors:** Marta Kazimierska, Marta Podralska, Magdalena Żurawek, Tomasz Woźniak, Marta Elżbieta Kasprzyk, Weronika Sura, Wojciech Łosiewski, Iwona Ziółkowska‐Suchanek, Joost Kluiver, Anke van den Berg, Natalia Rozwadowska, Agnieszka Dzikiewicz‐Krawczyk

**Affiliations:** ^1^ Institute of Human Genetics Polish Academy of Sciences Poznań Poland; ^2^ Institute of Bioorganic Chemistry Polish Academy of Sciences Poznań Poland; ^3^ Department of Pathology and Medical Biology University of Groningen, University Medical Center Groningen The Netherlands

**Keywords:** CRISPR/Cas9, high‐throughput screen, MYC, MYC target genes, transcription factor

## Abstract

The transcription factor MYC is a proto‐oncogene with a well‐documented essential role in the pathogenesis and maintenance of several types of cancer. MYC binds to specific E‐box sequences in the genome to regulate gene expression in a cell‐type‐ and developmental‐stage‐specific manner. To date, a combined analysis of essential MYC‐bound E‐boxes and their downstream target genes important for growth of different types of cancer is missing. In this study, we designed a CRISPR/Cas9 library to destroy E‐box sequences in a genome‐wide fashion. In parallel, we used the Brunello library to knock out protein‐coding genes. We performed high‐throughput screens with these libraries in four MYC‐dependent cancer cell lines—K562, ST486, HepG2, and MCF7—which revealed several essential E‐boxes and genes. Among them, we pinpointed crucial common and cell‐type‐specific MYC‐regulated genes involved in pathways associated with cancer development. Extensive validation of our approach confirmed that E‐box disruption affects MYC binding, target‐gene expression, and cell proliferation *in vitro* as well as tumor growth *in vivo*. Our unique, well‐validated tool opens new possibilities to gain novel insights into MYC‐dependent vulnerabilities in cancer cells.

## Graphical abstract

A MYC‐EBOX‐CRISPR library was used to disrupt MYC‐bound E‐boxes, which reduced MYC binding and affected the expression of target genes. The Brunello library was used to knock out protein‐coding genes. Overlap of the results from both CRISPR screens allowed for identification of MYC binding sites and adjacent target genes essential for growth of cancer cells *in vitro* and *in vivo*.

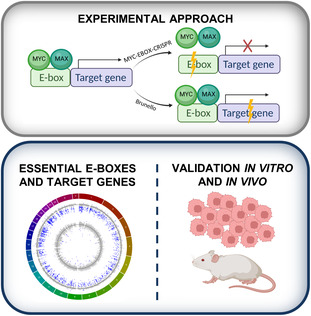

**Abbreviations**
(d)Cas9: (dead)CRISPR‐associated protein
bHLH‐LZ: basic helix–loop–helix‐leucine‐zipper family
BL: Burkitt lymphoma
ChIP‐seq: chromatin immunoprecipitation followed by sequencing
CML: chronic myelogenous leukemia
CRISPR: clustered regularly interspaced palindromic repeats
DMEM: Dulbecco's Modified Eagle's Medium
FC: fold change
GO: Gene Ontology
GSEA: Gene Set Enrichment Analysis
MAX: MYC‐associated factor X
PAM: protospacer adjacent motif
PBS: phosphate‐buffered saline
ROI: regions of interest
RPMI: Roswell Park Memorial Institute 1640 medium
sgRNA: single‐guide RNA
TF: transcription factor
TSS: transcription start site

## 1 Introduction

MYC is an oncogene broadly involved in many tumors. Due to amplifications, mutations, translocations, or posttranslational modifications, MYC is highly expressed in up to 70% of cancers [1]. The family of MYC transcription factors (TFs) consists of three members: C‐MYC, N‐MYC, and L‐MYC [2]. Among them, C‐MYC (further called MYC) has the strongest oncogenic potential and is widely deregulated in cancer, while N‐MYC and L‐MYC are mainly involved in neuroblastoma and lung cancer, respectively [3, 4, 5]. MYC regulates expression of genes associated with cell cycle, apoptosis, proliferation, and cellular differentiation, as well as strongly alters metabolism of cancer cells and stimulates ribosome and mitochondrial biogenesis [6, 7, 8, 9]. Altogether, this creates a strong dependency of cancer cells on MYC and it has been demonstrated that indeed MYC withdrawal leads to tumor regression [10, 11]. Thus, targeting MYC appears as an attractive strategy for cancer therapies. However, no clinically relevant MYC‐targeting therapies have been developed so far [12]. MYC is considered an ‘undruggable target’ due to its localization and activity in the nucleus and lack of an active site for interaction with small molecules [2, 4]. Therefore, there is a need to look for indirect approaches such as identification of essential MYC‐regulated genes, which may potentially expand the repertoire of novel antitumor therapies.

MYC is a TF belonging to the basic helix–loop–helix leucine zipper family (bHLH‐LZ) that creates heterodimers with the MYC‐associated factor X (MAX). The MYC/MAX complex recognizes and binds to E‐box motifs in DNA (canonical sequence 5′‐CACGTG‐3′), localized mainly in promoters and enhancers. However, MYC binding is not restricted to the E‐boxes. Moreover, these motifs can be also recognized by other members of the bHLH‐LZ family. Although MYC has been widely studied, its targetome is not fully known. Thousands of genes responding to MYC activation or inhibition have been identified, but it is difficult to distinguish direct and indirect targets [13, 14, 15, 16]. Chromatin immunoprecipitation with MYC antibody followed by sequencing (MYC‐ChIP‐seq) indicated thousands of MYC‐bound sites in the genome [13, 17, 18, 19, 20]. However, the set of MYC target genes varies among different cell types and developmental stages [21, 22]. This may be explained by the fact that MYC binds predominantly to already active promoters or enhancers and inactive genes remain silent [17]. While it was shown that disruption of even one gene, crucial for MYC‐dependent cancer development can be sufficient to decrease cancer cell growth [23], it is still not clear which MYC targets are essential for cancer cells. To date, a limited number of RNA interference screens for genes essential in MYC‐driven tumors have been performed. However, they either focused only on a limited set of genes or were performed in normal cells with forced MYC overexpression [24, 25, 26, 27]. This precludes their direct relevance to cancer cells. So far, thousands of MYC binding sites and regulated target genes have been identified; however, the question which of them are actually essential for MYC‐dependent cancer cells is still open.

In this study, we created a novel tool for the identification of essential MYC‐bound E‐boxes and target genes in MYC‐dependent cancer cells. We designed a sgRNA (single‐guide RNA) library for a genome‐wide disruption of MYC binding sites and conducted a high‐throughput screen in four MYC‐dependent cell lines: K562 (chronic myelogenous leukemia, CML), ST486 (Burkitt lymphoma, BL), HepG2 (hepatoblastoma), and MCF7 (breast cancer). Those cell lines overexpress MYC and strongly depend on its high levels [28, 29, 30, 31]. To allow identification of the relevant nearby genes regulated by the E‐boxes, in parallel, we utilized the Brunello library for genome‐wide knockout of protein‐coding genes. Overlapping data from both screens identified several known and novel MYC targets critical for those cells. Altogether, we established a unique, well‐validated tool to identify MYC‐regulated target genes relevant for growth of malignant cells, which opens the possibility to gain novel insights into MYC‐dependent vulnerabilities in cancer cells.

## 2 Materials and methods

### 2.1 Cell lines

The chronic myeloid leukemia cell line K562 (RRID:CVCL_0004; ordered from DSMZ, Braunschweig, Germany) and BL cell line ST486 (RRID:CVCL_1712; ATCC, Manassas, VA, USA) were maintained in Roswell Park Memorial Institute 1640 medium (RPMI, Lonza, Basel, Switzerland) supplemented with 10–20% fetal bovine serum (Sigma‐Aldrich, Saint Louis, MO, USA), 2 mm l‐glutamine (Biowest, Nuaille, France), and 1% penicillin/streptomycin (Biowest) in a 5% CO_2_ incubator at 37 °C. Hepatocellular carcinoma cell line HepG2 (RRID:CVCL_0027; DSMZ), breast cancer cell line MCF7 (RRID:CVCL_0031; ECACC, Porton Down, UK), and HEK293T cell line (RRID:CVCL_0063; DSMZ) used for lentiviral particle production were cultured in low glucose Dulbecco's Modified Eagle's Medium (DMEM, Lonza) supplemented as described above. In addition, medium for MCF7 cells was supplemented with 1× NEAA (Gibco, Waltham, MA, USA). Cell lines were authenticated by providers in the period of 3 years before the experiments. They were expanded and banked in our laboratory immediately upon receipt. After thawing, cells were cultured for experiments no longer than 3 months. Mycoplasma tests were routinely performed and confirmed that the cells were not contaminated.

### 2.2 Plasmids

The lentiCRISPR v2 (#52961) [32] and lentiCRISPR v2‐dCas9 (#112233) [33] vectors were purchased from Addgene (Watertown, MA, USA). The plasmids contain the coding sequence of *S. pyogenes* (d)Cas9 and a cloning site for the sgRNA, as well as a puromycin resistance gene allowing selection of transduced cells. pGreenpuro lentivectors (SBI, Mountain View, CA, USA), with MYC shRNAs and scrambled control, a kind gift of prof. Anke van den Berg [34], were used for experiments with MYC knockdown.

### 2.3 Design of the MYC‐EBOX‐CRISPR library

To design a library of sgRNAs for comprehensive genome‐wide targeting of MYC binding sites in cancer cells, we utilized publicly available MYC‐ChIP‐seq data from MCF7, K562, and HepG2 cells [35] and BL cell lines [19]. Genomic coordinates (hg19) of MYC‐ChIP peaks were obtained using UCSC Table Browser and from the published data [19]. Genomic intervals were concatenated and merged, and DNA sequences were retrieved using galaxy [36]. To avoid targeting coding exons, which could have resulted in disruption of the protein apart from the E‐box, sequences overlapping with coding exons (based on Ensembl hg19 annotation) were removed. In the resulting sequences, E‐box motifs were identified (canonical CACGTG, non‐canonical CACATG/CATGTG, and CACGCG/CGCGTG), and all sgRNAs targeting these E‐boxes were designed based on the presence of the PAM (protospacer adjacent motif) sequence (NGG or CCN) using an in‐house python script (https://github.com/tomaszwozniakihg/cas9_search_tool). The resulting sgRNAs were checked for off‐target binding using the cas‐ot script [37]. Only sgRNAs with at least three mismatches to the potential off‐targets or at least two mismatches including at least one in the seed region (nt 9–20) were retained. Later analysis using the CFD algorithm, which was not available at the time of library design, gave similar results. Only 81 sgRNAs included in the library had CFD scores lower than 30; they are marked in red in Table S1. The library also included 1000 non‐targeting sgRNAs as a negative control, and four sgRNAs against MYC as a positive control, all from the Brunello library. A list of all sgRNA oligonucleotides is provided in Table S1. File S1 contains .bed file with coordinates of targeted E‐boxes, which can be used in ucsc genome browser (hg19).

### 2.4 Cloning of the MYC‐EBOX‐CRISPR library and amplification of the Brunello library

Oligonucleotides containing the 20 nt sgRNA sequences flanked by the sequence from the lentiCRISPR_v2 vector TTTCTTGGCTTTATATATCTTGTGGAAAGGACGAAACACCG [20 nt sgRNA] GTTTTAGAGCTAGAAATAGCAAGTTAAAATAAGGCTAGTCCGT were synthesized by Twist Bioscience (San Francisco, CA, USA). Two nanogram of oligonucleotide pool was amplified with the oligo‐F and oligo‐R primers (Table S2) using the NEBNext High‐Fidelity Master Mix (New England Biolabs, Ipswich, MA, USA) in 20 × 25 μL PCR reactions. PCR program: 98 °C 30 s; (98 °C 10 s; 63 °C 10 s; 72 °C 15 s) × 7 cycles; 72 °C 2 min. PCR product was purified using QIAquick PCR Purification Kit (Qiagen, Hilden, Germany) and then extracted from an agarose gel with QIAquick Gel Extraction Kit (Qiagen). LentiCRISPRv2_puro vector was digested with the BsmBI restriction enzyme (New England Biolabs) and purified from an agarose gel. sgRNA MYC‐EBOX‐CRISPR library was cloned into lentiCRISPRv2_puro vector using the circular polymerase extension cloning (CPEC) method as described previously [38]. Briefly, 20 CPEC reactions were performed, each using 100 ng digested vector, 10.8 ng amplified oligos, and NEBNext High‐Fidelity Master Mix. PCR program: 98 °C 30 s; (98 °C 10 s; 72 °C 7 min) × 5 cycles; 72 °C 5 min. All PCR reactions were pooled and purified by isopropanol precipitation. Three hundred nanogram of the CPEC product was used for transformation of electrocompetent Endura cells (Lucigen, Middleton, WI, USA) according to the manufacturer's protocol. Fourteen electroporations were performed giving in total ~ 7.9 million colonies and resulting in ~ 170× coverage of the library. Bacteria were spread on 245 × 245 agar plates and grown for 14 h at 37 °C. Colonies were scraped off the plates, and plasmid DNA was isolated using Plasmid Plus Maxi Kit (Qiagen). The MYC‐EBOX‐CRISPR library was deposited in Addgene (#173195). Brunello library targeting all human protein‐coding genes [39] was purchased from Addgene (#73179). Fifty nanogram of the library was electroporated into Endura cells. Four electroporations were performed resulting in > 2500× coverage. Quality of the MYC‐EBOX‐CRISPR and Brunello libraries was verified by next‐generation sequencing on Illumina platform (BGI, Hong Kong).

### 2.5 Cloning of individual sgRNA constructs

For cloning of individual sgRNAs (Table S3) into the lentiCRISPRv2_puro and lentiCRISPRv2‐dCas9 vectors, sense and antisense oligos containing overhangs compatible with BsmBI sticky ends were synthesized by Genomed (Warsaw, Poland). Oligonucleotides were diluted in annealing buffer (10 mm Tris–HCl pH 8, 1 mm EDTA pH 8, 50 mm NaCl). Annealing was performed in a thermocycler under conditions: 95 °C 5 min, 95 °C (−1 °C/cycle) × 70 cycles. Annealed sgRNAs were ligated into the lentiCRISPRv2 and lentiCRISPRv2‐dCas9 vectors digested with BsmBI restriction enzyme at 1 : 5 vector : insert molar ratio with T4 DNA ligase (Invitrogen, Carlsbad, CA, USA). One microliter of ligation reaction was transformed into JM109 competent cells (Promega, Madison, WI, USA). Plasmid DNA from single colonies was isolated using Plasmid Plus Maxi Kit (Qiagen). Sequences of individual sgRNA constructs were confirmed by Sanger sequencing (Genomed).

### 2.6 Generation of lentiviral particles

For large‐scale production of lentiviral particles, ~ 7.5 million HEK293T cells were plated in a T75 flask 1 day prior to transfection. The next day, ~ 80% confluent cells were transfected with packaging plasmids psPAX (11.2 μg) and pMD2.G (7.5 μg), and MYC‐EBOX‐CRISPR or Brunello library plasmid (15 μg) using lipofectamine 2000 reagent (Invitrogen). One day after transfection, medium was replaced with 7.5 mL DMEM supplemented with 10% FBS. Two and 3 days post‐transfection, Brunello and MYC‐EBOX‐CRISPR lentiviral supernatants were filtered through a 0.45 μm filter and stored at −80 °C. For small‐scale production of lentiviral particles, 1 million HEK293T cells were plated per well on a 6‐well plate and transfected the next day with packaging plasmids psPAX (1.5 μg) and pMD2.G (1 μg), and lentiCRISPRv2 plasmid (2 μg) using calcium phosphate transfection method (Invitrogen). One day after transfection, medium was replaced with 1.1 mL DMEM supplemented with 10% FBS and lentiviral supernatant was collected as described above.

### 2.7 Determination of virus titer and cell transduction for screening

1.8–2.7 million cells were plated per well in a 12‐well plate and transduced with different amounts of virus. 4 mg·mL^−1^ polybrene was added, and cells were spun down in plates (33 °C, 1000 × *g*, 2 h). After spinfection, additional 1 mL of medium was added. Twenty‐four hours after transduction, cells were washed and plated out in four wells for each condition, two wells with 0.3–3 μg·mL^−1^ puromycin and two wells without puromycin. Medium with puromycin was changed after 3 days. After 4 days of selection, cells were counted and the percentage of surviving cells relative to cells not treated with puromycin was calculated. From this, we determined the amount of virus resulting in ~ 30% surviving cells, which implies that ~ 85% of transduced cells were infected by a single virus.

Approximately seventy‐eight million cells for the MYC‐EBOX‐CRISPR library and ~ 130 million cells for the Brunello library were transduced in duplicate with the amount of virus that results in ~ 30% transduced cells, in the same conditions as described above. After 4 days of selection with puromycin, (T0) part of the cells was collected for DNA isolation. Remaining cells were further cultured for 20 population doublings. At each passage, the amount of cells corresponding to a 500× coverage (24 million for MYC‐EBOX‐CRISPR, 38 million for Brunello library) were cultured in RPMI medium with 0.1–1 μg·mL^−1^ puromycin and collected at the final timepoint (T1).

### 2.8 Preparation of libraries for next‐generation sequencing of the plasmid pool or genomic DNA

sgRNA sequences from the MYC‐EBOX‐CRISPR and Brunello library plasmids were amplified in PCR reaction using High‐Fidelity MasterMix 2× (New England Biolabs) and primers containing Illumina adaptors, an 8 nt barcode specific for each library (reverse primer) and a variable length (9–18 nt) stagger sequence to increase library complexity (forward primer) [40] (Table S2). PCR products were pooled and purified using QIAquick PCR Purification Kit (Qiagen). Amplicons were analyzed on agarose gel and then extracted using QIAquick Gel Extraction Kit (Qiagen). Quality check and quantification of libraries were performed by qPCR using KAPA Library Quantification Kit (Roche, Basel, Switzerland).

Genomic DNA collected from cells infected with MYC‐EBOX‐CRISPR and Brunello libraries was isolated using GENTRA Puregene Kit (Qiagen). PCR was performed to amplify the sgRNA sequences integrated into genomic DNA. DNA from 23 million cells (MYC‐EBOX‐CRISPR) and 38 million cells (Brunello) was amplified in 80–120 (MYC‐EBOX‐CRISPR) and 130–210 (Brunello) individual 50 μL PCR reactions per sample with 3–3.5 μg DNA input as described above. PCR products were purified, analyzed on agarose gel, pooled based on band intensities, and extracted from gel as described above. Determination of quality and quantification of libraries was performed by qPCR (Kapa Library Quantification Kit).

### 2.9 NGS and data analysis

NGS was performed on Illumina X‐Ten platform at BGI (Hong Kong). Reads were trimmed to remove adaptor sequences and split based on barcodes for individual samples. Number of reads obtained for each sample is given in Table S4. For sgRNA enumeration, raw reads were processed with a Python script [40]. Only sgRNAs with no mismatches were counted. Fold change (FC) and *P*‐values for each gene and E‐box were calculated with DeSeq2. Read counts for all sgRNAs for a given E‐box or gene were summed up, and DeSeq2 analysis was performed, which includes the estimation of size factors, the variance stabilization using a parametric fit, and a Wald test for difference in log2 FCs between the untreated and treated data [41]. Adjusted *P*‐value 0.001 was used as a cut‐off for identification of significantly depleted or enriched E‐boxes (MYC‐EBOX‐CRISPR library) and genes (Brunello library).

### 2.10 Determination of CRISPR/Cas9 editing efficiency by TIDE

To confirm CRISPR/Cas9 disruption of selected E‐boxes, DNA was isolated from K562 cells on day 7 after transduction with individual sgRNAs targeting the selected E‐boxes. Genomic regions of ~ 500–800 bp flanking E‐box sequences were amplified by PCR (primer sequences in Table S2). Amplicons were sequenced using the Sanger method (Genomed) and analyzed with TIDE calculator (https://tide‐calculator.nki.nl) [42], using indel size range of 50.

### 2.11 RNA isolation and qRT‐PCR

Total RNA was isolated from K562 cells using Quick‐RNA™ Miniprep Kit (Zymo Research, Irvine, CA, USA). cDNA was synthesized from 500 ng RNA using QuantiTect® Reverse Transcription Kit (Qiagen). qPCR on 5 ng cDNA was performed on CFX96 Touch qPCR System (BioRad, Hercules, CA, USA) using PowerUp SYBR Green Master Mix (Applied Biosystems, Waltham, MA, USA). Expression was normalized to TBP. All experiments were conducted in two independent biological replicates, each with three technical replicates.

### 2.12 Growth assay

Growth assay was performed using CellTiter‐Glo® Luminescent Cell Viability Assay (Promega). 1*10^3^ K562 cells infected with sgRNA lentiviral constructs were plated in triplicate on 96‐well plates. One hundred microliter Cell‐Titer Glo reagent diluted 1 : 2 in PBS was added per well after 1 h (baseline level), 48 and 96 h. The luminescent signal was measured using a GloMax microplate reader (Promega). Experiments were performed in three independent biological replicates. Growth rate was calculated at 48 and 96 h relative to the 1 h measurement.

### 2.13 Chromatin immunoprecipitation assay

10 m of K562 cells infected with sgRNAs targeting selected E‐box sequences were fixed to crosslink DNA with chromatin‐associated proteins according to the Active Motif protocol. Briefly, cells were fixed for 20 min by adding 1/10 volume of 37% formaldehyde solution and then neutralized by adding 1/20 volume of 2.5 m glycine. Next, K562 cells were washed using PBS‐Igepal, snap‐frozen on dry ice, and stored at −80 °C. Chromatin immunoprecipitation with anti‐MYC antibody (sc‐764, Santa Cruz, Dallas, TX, US) followed by qPCR was performed by Active Motif (La Hulpe, Belgium).

### 2.14 Luciferase reporter assay

To confirm that sequences containing selected E‐boxes drive transcription in an MYC‐dependent way, we conducted a luciferase reporter assay. MYC binding sites as defined by peaks from MYC‐ChIP‐seq data were amplified by PCR from DNA of K562 cells. For chr11_BS79, the amplified sequence was shorter to omit two other non‐essential E‐boxes present in the peak. Primers contained sequences to create overhangs for XhoI and SacI restriction sites (Table S2) for cloning into the pGL4.23 vector, upstream of the firefly luciferase gene under minimal promoter (#E8411, Promega). K562 cells were transduced with MYC shRNAs or scrambled control. After selection with puromycin for 4 days, 1 × 10^5^ cells were co‐transfected using Lipofectamine LTX with 500 ng pGL4.23 vector with E‐box constructs and 5 ng pRL‐SV40 vector containing Renilla luciferase (#E2231, Promega). Twenty‐four hours after transfection, cells were lysed and luminescence was measured using Dual‐Luciferase Reporter Assay on GloMax (Promega). Firefly luminescence was normalized to Renilla. The experiments were performed in three independent biological replicates, each with triplicate transfection.

### 2.15 Gene ontology, gene set enrichment analysis, and TCGA data

For each E‐box, the nearest genes with transcription start site (TSS) within 50 kb both upstream and downstream were retrieved using Galaxy. Gene Ontology analysis was conducted using DAVID Functional Annotation Tool v6.8b [43, 44] on (a) genes marked as essential in the Brunello screen and (b) a subset of at least twofold depleted genes within 50 kb of essential E‐boxes from MYC‐EBOX‐CRISPR screen. Preranked Gene Set Enrichment Analysis (GSEA) [45, 46] was performed on log2 FC values of all genes in the Brunello library. To complement this analysis and reveal processes in which genes regulated by essential E‐boxes are involved, we have taken a subset of genes from the Brunello screen which were near at least two‐fold depleted E‐boxes. Hallmark (H) and curated (C2) gene sets v7.4 were used for analysis. In addition, we analyzed expression of selected genes in tumor and normal tissues using data from the UALCAN portal and explored potential relationship with survival [47, 48].

### 2.16 Tumor xenografts

The experiments were carried out in 2‐month‐old NOD‐SCID mice (NOD, CB17‐Prkdcscid/NCrCrl) bred and housed in Animal Facility in IHG PAS, Poznan, Poland. Animals were provided sterilized food and water *ad libitum* and were maintained on a regular 12‐h day/night cycle at no more than five adult animals per individually ventured cage. The Local Ethical Committee for Animal Research at Poznan University of Life Sciences approved the protocol for the experiments performed in the mice (LECfAR decision 58/2020). All animal experiments were performed under relevant guidelines and regulations according to 3R rules. The experimental groups were designed to include the same ratio of male/female individuals. HepG2 cells stably expressing firefly luciferase were transduced with control non‐targeting sgRNA or sgRNAs targeting E‐boxes: chr1_BS1363_CACAATG, chr11_BS79_CGCGTG, and chr18_BS691_CATGTG. 1 × 10^6^ cells per dose/2 × 10^6^ per mouse cells were resuspended in the mixture of medium and Matrigel (Merck, Darmstadt, Germany) and injected subcutaneously into the right and left flank of each mouse. Mice were randomly classified into four groups (*n* = 4–8 tumors per group). *In vivo* bioluminescence imaging was performed every 7 days for 5 weeks. Mice were anesthetized with 5% isoflurane and maintained with 2% isoflurane during imaging procedures. Luciferase‐based bioluminescence imaging was performed with an IVIS Lumina LT imaging system (Perkin Elmer, Waltham, MA, USA) equipped with a camera box and warming stage. Following intraperitoneal injection of 150 μL of IVISbrite D‐Luciferin Potassium Salt Bioluminescent Substrate (XenoLight; Perkin Elmer) dissolved in phosphate‐buffered saline (PBS), mice were immediately imaged with sequential five numbers of segments in every 2 min delay with 2 s exposures. Images were captured, and bioluminescence intensity was quantitated using living image 3.2 acquisition and analysis software (Caliper Life Sciences, Hopkinton, MA, USA). Total flux values were determined by drawing regions of interest (ROI) of identical size over each mouse and are presented in photons (p) per second (s). All animals were sacrificed after 5 weeks of inoculation. Tumor volume was determined by a vernier caliper and calculated by the formula: volume = (width^2^ × length)/2.

## 3 Results

### 3.1 Generation of the MYC‐EBOX‐CRISPR library for a genome‐wide disruption of MYC binding sites

**Fig. 1:**
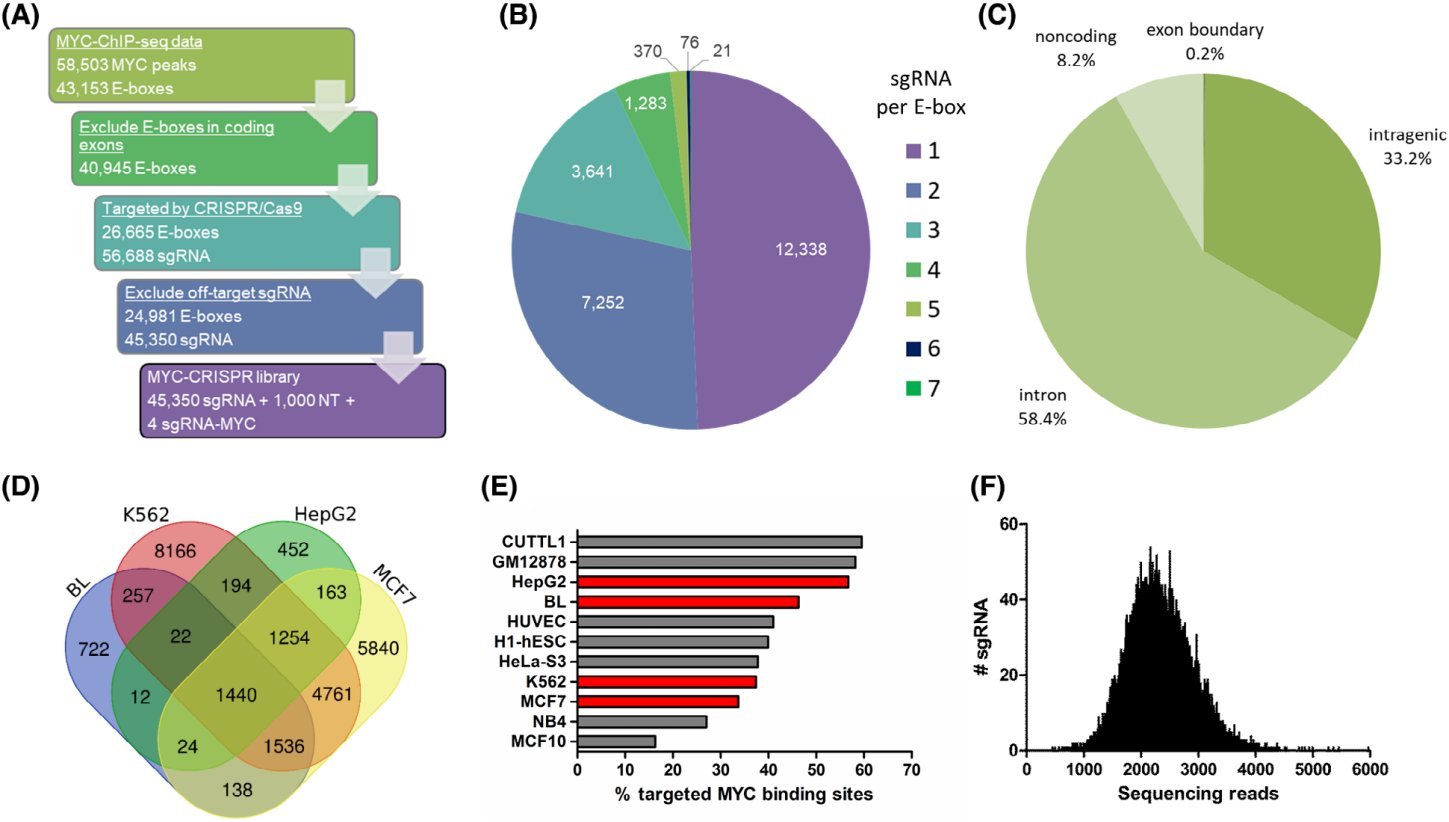
Design and generation of the MYC‐EBOX‐CRISPR library for genome‐wide disruption of MYC binding sites. (A) Library was designed based on publicly available MYC‐ChIP‐seq data in MYC‐dependent K562, MCF7, HepG2, and Burkitt lymphoma (BL) cell lines. After excluding E‐boxes in coding exons, all possible sgRNAs targeting remaining E‐boxes were designed, based on the presence of PAM sequence. sgRNAs with predicted off‐target binding were filtered out. The final library contains 43 350 sgRNAs targeting E‐boxes, 1000 non‐targeting (NT) sgRNAs as a negative control, and four sgRNAs targeting MYC as a positive control. (B) Number of sgRNA constructs per E‐box. (C) Genomic location of E‐boxes targeted by the library. (D) Overlap of targeted E‐boxes for selected cancer cell lines. (E) Percentage of MYC binding sites targeted by the MYC‐EBOX‐CRISPR library in various cell lines, based on available MYC‐ChIP‐Seq data. In red, are cell lines for which the library was designed. (F) Distribution of sgRNA constructs in the MYC‐EBOX‐CRISPR plasmid library determined by NGS. All sgRNAs were present in the library.

We confirmed that cell lines selected for this study: K562, ST486, HepG2, and MCF7 express high levels of MYC and depend on MYC for their growth (Fig. S1). From publicly available MYC‐ChIP‐seq data in MYC‐dependent K562, MCF7, HepG2 [35], and BL cell lines [19], we obtained 58 503 MYC binding sites, which contained 43 153 E‐box motifs. 2208 of them were located in the coding exons and were excluded to prevent effects due to disruption of the protein. Based on the presence of PAM sequence, we designed 56 688 unique sgRNAs targeting the remaining 26 653 E‐boxes (65.1%). After excluding sgRNAs with predicted off‐target binding, we finally obtained 45 350 sgRNAs targeting 24 981 E‐boxes (61%; Fig. 1A, Table S1). Half of the E‐boxes were targeted by more than one sgRNA (Fig. 1B). The majority of E‐boxes were located in introns (58.4%) or intergenic regions (33.2%; Fig. 1C). Sixty‐one percent of the E‐boxes targeted by our MYC‐EBOX‐CRISPR library were bound by MYC only in one cell line, while 6% of the E‐boxes were commonly bound by MYC in all four cell lines (Fig. 1D). This is in line with the high cell‐type specificity of MYC targets. Although the MYC‐EBOX‐CRISPR library was designed based on the data from selected four cell lines, analysis of available MYC‐ChIP‐seq data from 11 cell lines revealed that 16–60% MYC binding sites were targeted by the library sgRNAs (Fig. 1E). This indicates that the MYC‐EBOX‐CRISPR library can be widely applied for studies in various cell lines.

Next‐generation sequencing revealed high quality of the cloned MYC‐EBOX‐CRISPR library. All sgRNAs were present and the skew ratio of 90^th^ to 10^th^ percentile was only 1.76, indicating a uniform representation of all constructs in the library (Fig. 1F). The quality of the amplified Brunello library also conformed to the recommended requirements (Fig. S2). Thus, we generated a high‐quality MYC‐EBOX‐CRISPR library for genome‐wide targeting of MYC‐bound E‐boxes that can be universally used in various cell lines.

### 3.2 High‐throughput screen for functional MYC binding sites and target genes essential for growth of cancer cells

**Fig. 2:**
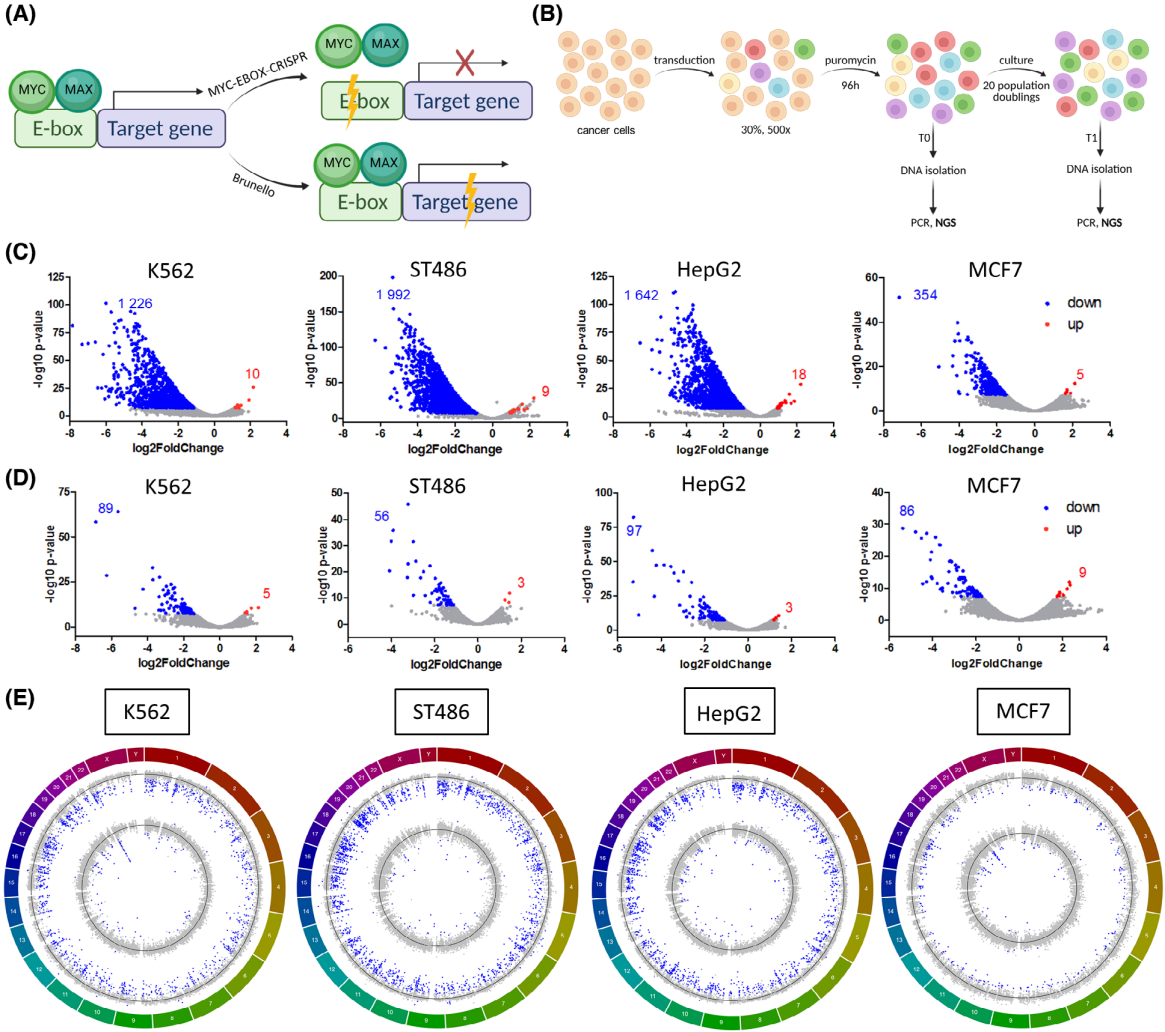
High‐throughput screen with MYC‐EBOX‐CRISPR and Brunello libraries. (A) Experimental approach: (1) MYC‐EBOX‐CRISPR library to destroy E‐box sequences, disrupt MYC binding, and its effect on target‐gene expression (2) Brunello library for genome‐wide gene knockout. (B) Scheme of the high‐throughput screen in cancer cells with MYC‐EBOX‐CRISPR and Brunello libraries. (C) DESeq2 analysis revealed essential genes in Brunello library and (D) essential E‐boxes in MYC‐EBOX‐CRISPR (depleted genes and E‐boxes in blue, enriched genes and E‐boxes in red). (E) Circos plots showing log2 fold change (FC) values for genes in Brunello screen (outer circle) and E‐boxes in MYC‐EBOX‐CRISPR screen (inner circle) across the chromosomes. Blue dots indicate genes and E‐boxes significantly (*P*_adj_ < 0.001) depleted or enriched, black lines denote log2FC = 0. (A and B) were created using BioRender.

To identify essential MYC binding sites and target genes, we conducted a genome‐wide high‐throughput CRISPR/Cas9 screen with the MYC‐EBOX‐CRISPR library (46 354 sgRNA) targeting E‐boxes and the Brunello library (77 441 sgRNA) [39] targeting protein‐coding genes (Fig. 2A). Four cancer cell lines were infected in duplicate aiming at ~ 500× coverage of each sgRNA in both libraries and a 30% transduction efficiency. The obtained values are shown in Table 1. Cells were collected at T0 (after puromycin selection) and T1 (after 20 population doublings), and the abundance of sgRNA constructs was determined by NGS (Fig. 2B, Tables S5 and S6). Almost none of the non‐targeting sgRNAs was depleted ≥ twofold in both replicates and two to four sgRNAs targeting MYC showed consistently decreased abundance at T1. 9112–11 970 sgRNAs from the Brunello library and 517–3034 sgRNAs from the MYC‐EBOX‐CRISPR library were consistently ≥ twofold depleted in both replicates (Fig. S3). Considering the combined effect of all sgRNAs targeting a given gene or E‐box, using DESeq2 algorithm, 354–1992 genes (Table S7) and 56–97 E‐boxes (Table S8) were identified as essential for growth of selected cancer cells (*P*_adj_ < 0.001), while 3–9 E‐boxes and 5–18 genes were significantly enriched (Fig. 2C,D).

**Table 1:** Performance of the screen in MYC‐dependent cancer cell lines conducted in duplicate.

	K562		ST486		HepG2		MCF7	
	Transduction efficiency	Coverage	Transduction efficiency	Coverage	Transduction efficiency	Coverage	Transduction efficiency	Coverage
MYC‐EBOX‐CRISPR #1	32%	540×	26.2%	435×	29%	485×	30%	500×
MYC‐EBOX‐CRISPR #2	26.9%	455×	32.1%	535×	33.3%	560×	25.4%	425×
Brunello #1	23%	390×	37.5%	625×	26.2%	440×	24.1%	405×
Brunello #2	24.5%	415×	34.6%	575×	24.1%	405×	23.7%	400×

Initial analysis in K562 cell line revealed 152 essential E‐boxes. Forty percent of them were localized on chromosome 22, near the breakpoint in *BCR* involved in the t(9;22) translocation (Fig. 2E). Hits observed in this region are most likely not caused by targeting essential genes but due to massive CRISPR/Cas9‐mediated DNA cleavage within this tandemly amplified region in K562 cells, as observed previously [49]. Indeed, an orthogonal approach with dCas9, which does not induce DNA cleavage but blocks E‐box sites to prevent MYC binding, demonstrated no effect on K562 cell growth for two sgRNAs from chromosome 22q11 (Fig. S4A). Therefore, E‐boxes from the amplified region on 22q11 and 9q34 were excluded from further analysis.

Analysis of essential E‐boxes revealed that 20–32% were localized close to genes essential for cancer cells, as identified in our screens with the Brunello library. Moreover, 42–49% of adjacent genes are well‐known MYC‐regulated targets (Table S8).

Thus, our CRISPR/Cas9 screen revealed known and novel MYC‐dependent vulnerabilities in the studied cancer cells.

### 3.3 Common and cell‐type‐specific MYC‐regulated processes

**Fig. 3:**
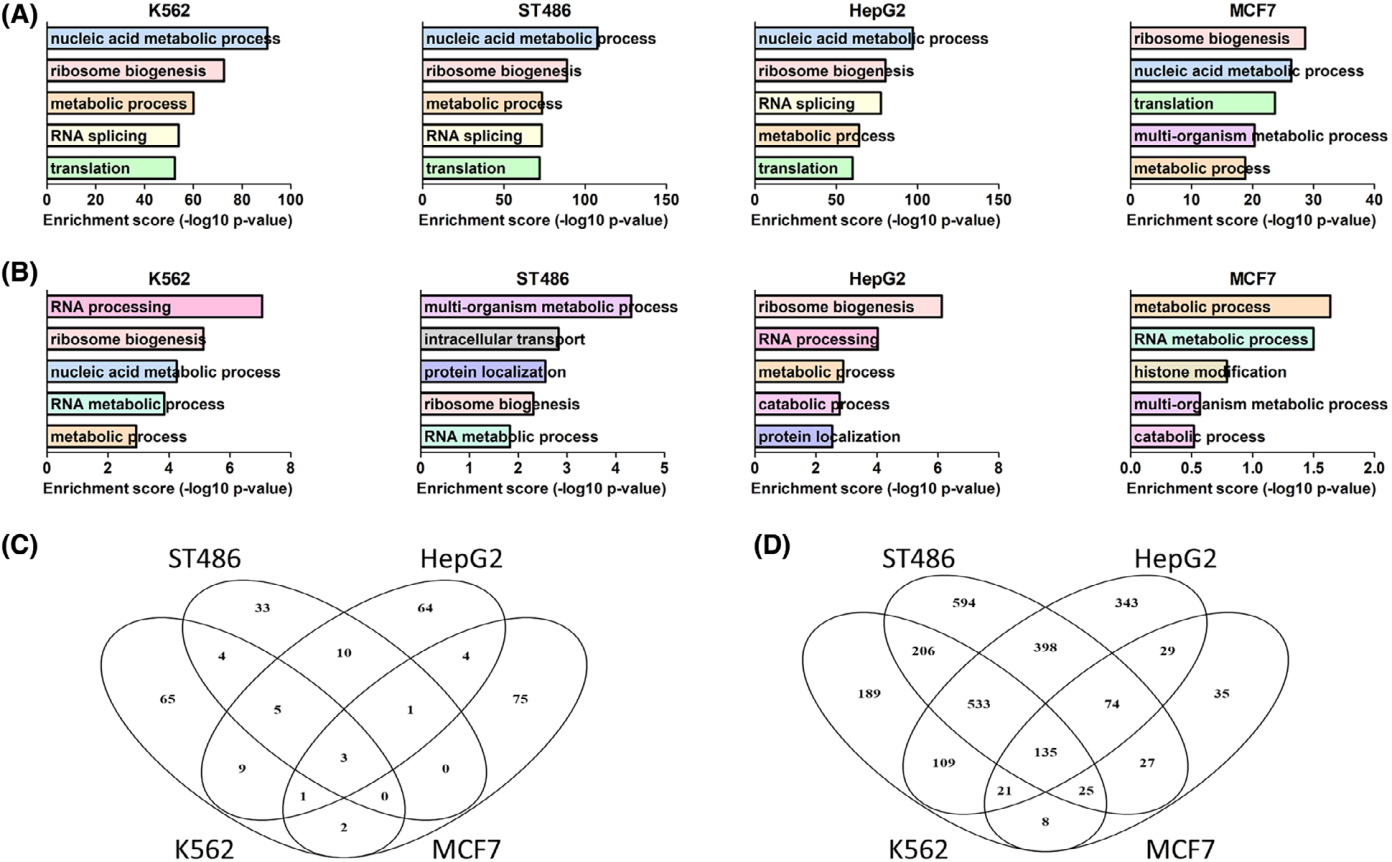
Essential MYC‐regulated processes and pathways. (A) Top five Gene Ontology (GO) terms for essential genes from Brunello library. (B) Top five GO terms for genes localized up to 50 kb from essential E‐boxes. (C) Overlap of essential E‐boxes from MYC‐EBOX‐CRISPR library. (D) Overlap of essential genes from Brunello library.

To determine main functions of essential and MYC‐regulated genes identified in our high‐throughput screens, we conducted GO and GSEA analyses. Genes essential in the Brunello screen were involved in several GO processes common for all cell lines, such as metabolism, ribosome biogenesis, metabolism of nucleic acids, splicing, and translation (Fig. 3A). GSEA results revealed very similar processes, majority of which were shared between cell lines. In addition, some cell‐specific processes emerged, such as DNA repair in K562, aminoacyl tRNA biosynthesis in ST486, oxidative phosphorylation in HepG2, and cell cycle in MCF7 (Table S9). On the contrary, genes localized near depleted E‐boxes showed a more diverse spectrum of processes, reflecting the limited overlap from the screen and indicating involvement of cell line‐specific processes regulated by MYC. We identified GO processes such as metabolism and ribosome biogenesis but also histone modifications, protein localization, RNA processing, and metabolism (Fig. 3B). GSEA for genes nearby E‐boxes highlighted translation, ribosome biogenesis, RNA processing, and tumor invasiveness as processes common for all cell lines. Cell‐type‐specific processes were much more prevalent and diverse, with no particular predominant terms emerging for each cell line (Table S10). Interestingly, REACTOME_REGULATION_OF_EXPRESSION_OF_SLITS_AND_ROBOS was a recurrently enriched gene set in all cell lines, in Brunello as well as MYC‐EBOX‐CRISPR results. SLIT/ROBO pathway is involved in axon guidance and cell migration, but it has been also implicated in tumor growth, migration, angiogenesis, and microenvironment [50].

Overlap of essential E‐boxes for four cancer cell lines revealed only three (1%) common E‐boxes (Fig. 3C): chr1_BS1363_CACAATG with neighbor genes *MECR* and *PTPRU*, chr11_BS79_CGCGTG localized near *RPLP2* and *PIDD1*, and chr18_BS691_CATGTG adjacent to *RBFA* and *TXNL4A*. Ten E‐boxes (3%) overlapped in three out of four cell lines, while majority of E‐boxes (59–87%) were essential only in one cell line. Detailed snapshots of exemplary common and specific E‐box loci are provided in Figs S5 and S6. Crucial genes identified in the Brunello screen showed greater overlap, with 135 (5%) genes common for all cell lines and 788 (29%) in three out of four, and only 10–30% cell‐type‐specific genes (Fig. 3D).

Due to low number of genes depleted in the Brunello screen and located near essential E‐boxes specific for each cell line, GO and GSEA analyses did not reveal any significant terms. However, we noticed some interesting processes, such as transcription, RNA processing, MAPK cascade, DNA repair, replication, translation, and protein transport or ubiquitination (Table S11). To further explore the potential relevance of those genes, we analyzed their expression in liver and breast cancer and determined their potential association with survival using The Cancer Genome Atlas (TCGA) data. Fourteen out of 18 depleted (log2FC < −1) genes near essential E‐boxes in HepG2 cells were significantly and > 1.5‐fold overexpressed in liver tumors compared with normal tissue. Moreover, high expression of 12 out of 18 genes was associated with significantly worse survival (Table S11). This highlights the relevance of E‐boxes and genes identified in our screen in patient samples. However, in breast cancer samples only four out of 17 genes were significantly and > 1.5‐fold overexpressed, and only one gene—ZMYND8—was associated with patient survival.

### 3.4 Validation of the approach

To validate the results of the screen and confirm robustness of our approach for identification of MYC‐dependent vulnerabilities in cancer cells, we focused on the top 10 most significantly depleted E‐boxes in K562 cells. Of these, we included for validation six that were located nearby a protein‐coding gene that was at least fourfold depleted in Brunello screen (chr3_BS897_CATGTG, chr11_BS79_CGCGTG, chr11_BS2113_CACATG, chr13_BS121_CGCGTG, chr17_BS377_CACGTG, and chr19_BS2255_CACATG), and two E‐boxes adjacent to long noncoding RNA (lncRNA) genes (chr10_BS212_CACATG and chr2_BS1664_CACGTG). The remaining two of the top 10 E‐boxes were in vicinity of non‐essential genes and were not considered for validation. In addition, we also included for validation a non‐essential E‐box, chr17_BS377_CGCGTG, which was located 12 nt downstream of chr17_BS377_CACGTG (Fig. S7), to gain further insight into MYC regulation at this locus.

**Fig. 4:**
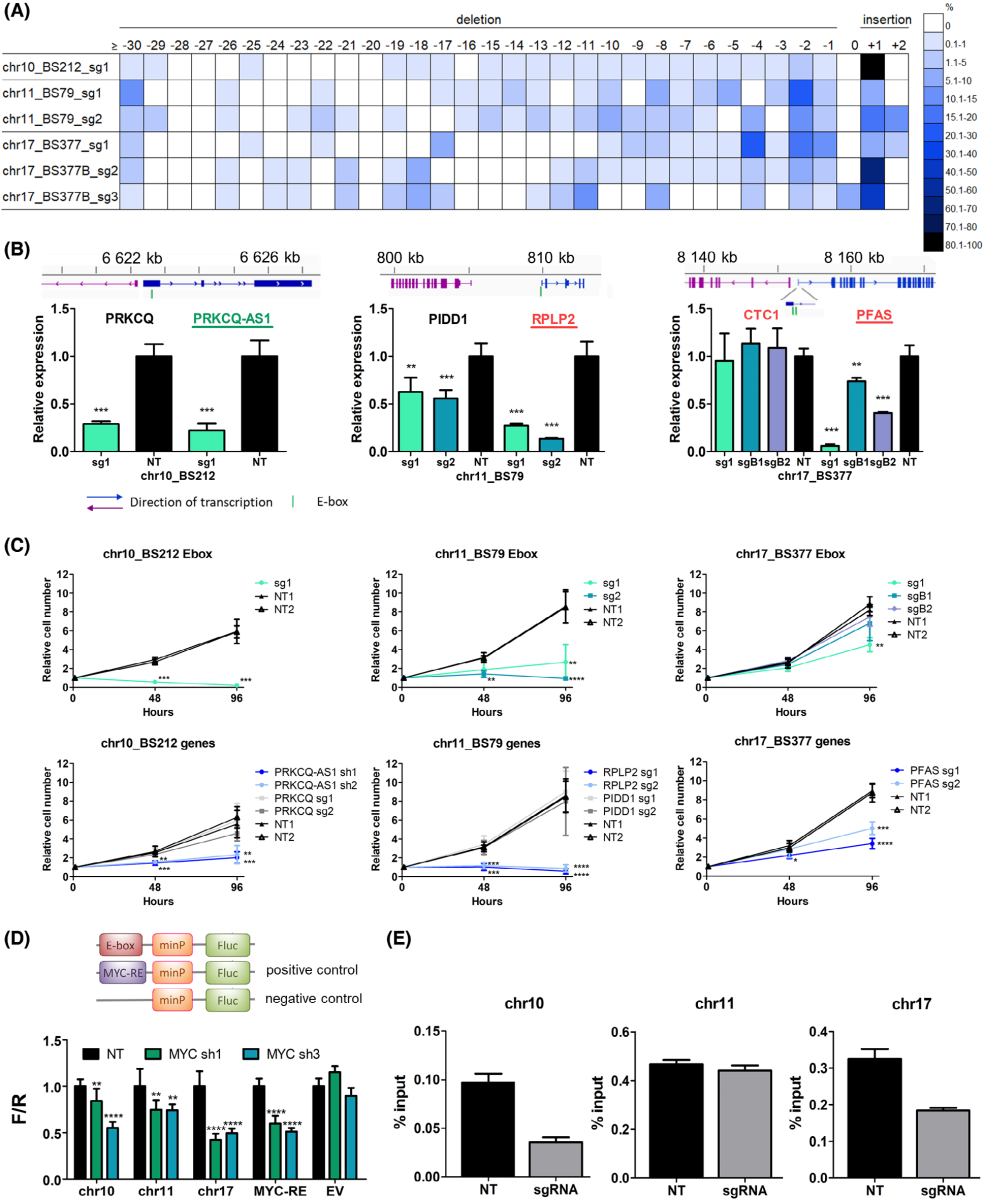
Validation of selected E‐boxes and target genes. (A) Efficiency of disruption of selected E‐boxes and the spectrum of mutations introduced by individual sgRNAs, demonstrated by TIDE analysis. Size distribution of introduced indels ranged from ≤ −30 to +2 bp. Colors indicate percentage of sequences with a given indel size. (B) qRT‐PCR analysis of genes adjacent to selected E‐boxes upon CRISPR/Cas9 disruption of E‐box sequences. Known MYC‐regulated genes are underlined; genes essential or at least fourfold depleted in Brunello screen are in red. Noncoding genes are in green. Despite the name ‘antisense’, PRKCQ‐AS1 does not overlap with PRKCQ. NT—average of two non‐targeting (negative control) sgRNAs. Mean and SD of two independent experiments, each performed in triplicate, are shown. **, *P* < 0.01; ***, *P* < 0.001, Student's *t*‐test. (C) Cell viability upon disruption of selected E‐boxes and knockout of adjacent genes was measured using CellTiter‐Glo assay at three time points: 0, 48, and 96 h. Shown are mean values and SD from three independent experiments, each performed in triplicate. *, *P* < 0.05; **, *P* < 0.01; ***, *P* < 0.001; ****, *P* < 0.0001, Student's *t*‐test. (D) Luciferase reporter assay for selected E‐boxes upon MYC knockdown with shRNA. Decreased luminescence signal was observed for all E‐boxes in MYC‐shRNA samples vs. NT control. **, *P* < 0.01; ****, *P* < 0.0001, Student's *t*‐test. Mean and SD of three independent experiments, each performed in triplicate, are shown. EV, empty vector, negative control; MYC‐RE, MYC responsive element, positive control. (E) MYC‐ChIP‐qPCR analysis of MYC binding upon E‐box disruption. Cells were infected with sgRNAs targeting selected E‐boxes. MYC binding was decreased for chr17_BS377 and for chr10_BS212 but not for chr11_BS79. Mean and SD from three replicates are shown.

Cells were transduced with individual sgRNAs targeting selected E‐boxes. First, we checked whether our approach allows for efficient disruption of E‐box motifs. TIDE analysis confirmed DNA editing with > 90% efficiency for all sgRNAs. The spectrum of mutations varied between individual constructs, with small 1–2 nt indels being most prevalent (Fig. 4A, Fig. S8A).

Next, we confirmed that for six out of eight selected E‐boxes, expression of adjacent genes was significantly affected (Fig. 4B, Fig. S8B). In some instances, expression of both genes nearby an E‐box was altered, while in others, one of the genes was not affected at all or to a lesser extent. Interestingly, for chr17_BS377 we observed strong downregulation of *PFAS* when targeting the essential CACGTG E‐box (sg1), but much weaker effect for the non‐essential CGCGTG E‐box (sgB1 and sgB2). For E‐box chr2_BS1664, we could not reliably detect expression of the adjacent lncRNAs, neither in control nor in CRISPR/Cas9‐edited samples. For chr11_BS2113, the closest gene was *PRKRIR* located > 50 kb, and we did not observe an impact on its expression. Thus, disruption of E‐boxes with CRISPR/Cas9 modulates expression of target genes and can indicate genes regulated by MYC within a given locus.

We further validated the effect on cell growth observed in high‐throughput screens for three E‐boxes with the strongest effect on expression of adjacent genes: chr10_BS212, chr11_BS79, and chr17_BS377. We conducted growth assays in K562 cells transduced with sgRNAs for E‐box disruption and knockout of adjacent genes, and shRNAs for knockdown of the lncRNA PRKCQ‐AS1, which was not included in the Brunello screen. For all sgRNAs targeting chosen E‐boxes, we observed a significant decrease in cell growth, consistent with the results of the screen. We confirmed that within chr17_BS377, only the CACGTG E‐box targeted by sg1 is essential for K562 cell growth. Moreover, knockout/knockdown of genes adjacent to each E‐box also significantly reduced cell growth, in line with the effect observed in the Brunello screen (Fig. 4C). Although both adjacent genes showed decreased expression after disruption of chr10_BS212 and chr11_BS79, only one of each pair was essential for cell growth. Combining the outcome of E‐box disruption on expression of adjacent genes with the effect of individual genes' knockout on cell growth allowed us to pinpoint the MYC targets relevant for the cell growth.

To confirm direct MYC binding and regulation of transcription, we performed a luciferase reporter assay for the three selected E‐boxes. We observed decreased luminescent signal for all three MYC binding sites upon MYC knockdown (Fig. 4D). This indicates that binding of MYC to these sequences results in MYC‐dependent transcription. Moreover, MYC‐ChIP in cells transduced with sgRNAs targeting two of the selected E‐boxes confirmed decreased MYC binding to chr17_BS377 and chr10_BS212 as compared to non‐targeting control sgRNA. Disruption of chr11_BS79 did not affect the strength of MYC binding (Fig. 4E).

As an alternative approach, to further validate the importance of MYC binding, we utilized dCas9 to block the E‐box sequences rather than to disrupt them when using WT Cas9. RT‐qPCR in cells transduced with dCas9 and sgRNAs targeting chr10_BS212, chr11_BS79, and chr17_BS377 showed the same pattern of gene expression as for WT Cas9 (Fig. S4A). In addition, the effect on cell growth for chr11_BS79 and chr17_BS377 was similar with dCas9 and WT Cas9 (Fig. S4B), while we did not notice change in K562 growth for the sgRNA targeting chr10_BS212. These results further confirm that disturbing MYC binding at these positions is causative for the observed effects on expression of target genes and cell growth.

Three E‐boxes included in the validation in K562 cells were also essential in ST486 cells: chr3_BS897_CATGTG, chr10_BS212_CACATG, and chr11_BS79_CGCGTG. We confirmed decreased cell growth and deregulated expression of nearby genes upon targeting of those E‐boxes in ST486 cells (Fig. S9).

Altogether, we confirmed that our approach allows for efficient disruption of E‐box motifs, which results in decreased MYC binding and affects expression of target genes.

### 3.5 E‐box disruption inhibits tumor growth *in vivo* in a mouse xenograft model

**Fig. 5:**
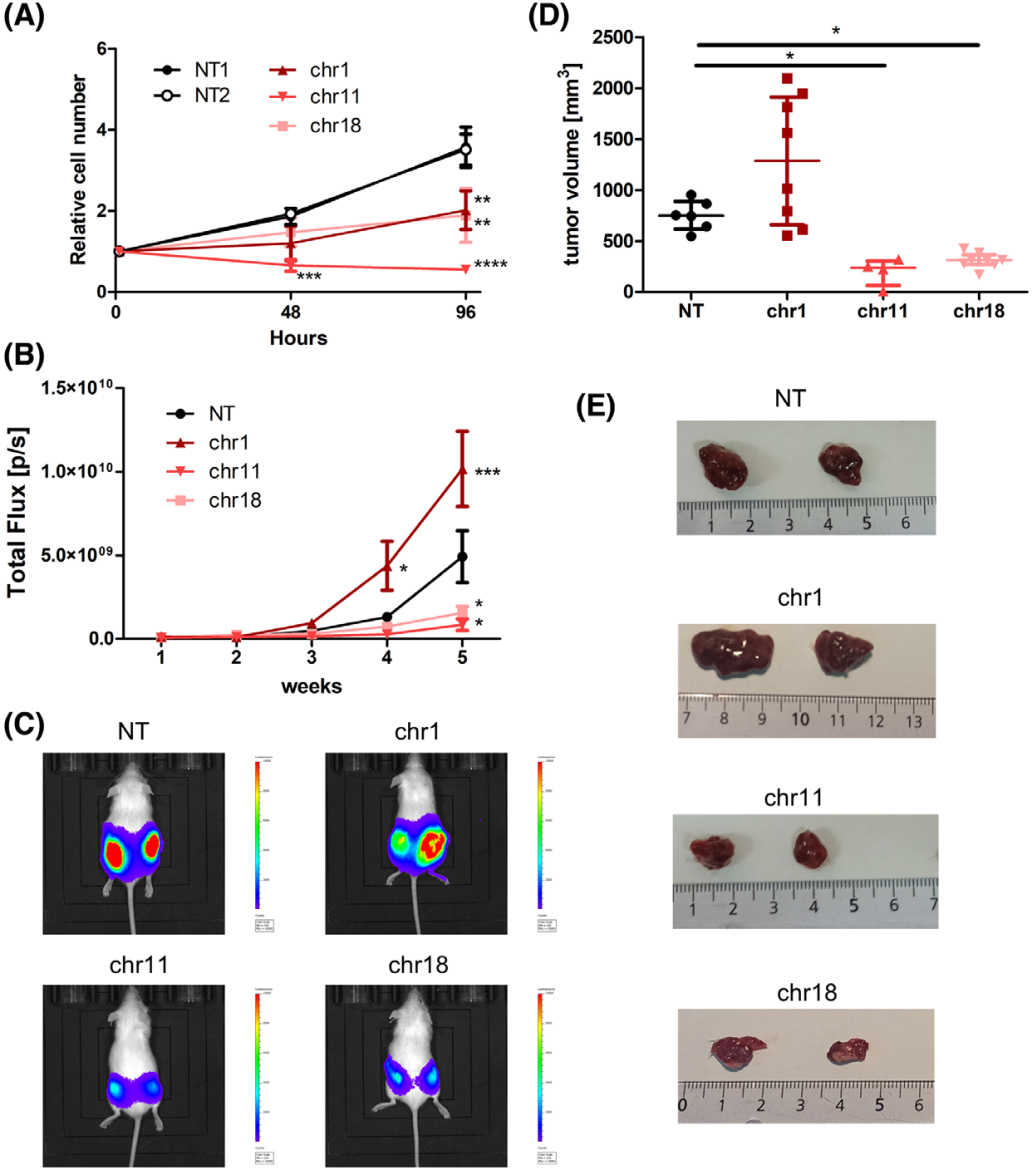
E‐box disruption inhibits tumor growth *in vivo*. HepG2 cells transduced with a non‐targeting (NT) control sgRNA or sgRNAs targeting E‐boxes on chr1, chr11, and chr18 were subcutaneously injected into NOD/SCID mice (NT *n* = 6 tumors; chr1 *n* = 8; chr11 *n* = 4; chr18 *n* = 8). (A) Confirmation of the decreased growth of HepG2 cells *in vitro* upon targeting selected E‐boxes. Shown are mean values and SD from three independent experiments, each performed in triplicate. *, *P* < 0.05; **, *P* < 0.01; ***, *P* < 0.001; ****, *P* < 0.0001, Student's *t*‐test. (B) Luciferase‐based bioluminescence imaging of tumors over 5 weeks, mean and SEM. *, *P* < 0.05; ***, *P* < 0.001, 2‐way ANOVA. (C) Representative images of luciferase‐based bioluminescence imaging on Week 5. (D) Volume of tumors excised from mice, median with interquartile range. *, *P* < 0.05, Kruskal–Wallis test with Dunn's post‐test. (E) Representative images of excised tumors [cm].

To validate the growth inhibitory effect *in vivo*, we established bioluminescent xenografts of HepG2 cells transduced with sgRNAs targeting the three common E‐boxes essential in all cell lines: chr1_BS1363_CACAATG, chr11_BS79_CGCGTG, and chr18_BS691_CATGTG, and the non‐targeting control. We chose HepG2 cells based on the feasibility of establishing the xenograft model. First, we confirmed the negative effect of targeting those E‐boxes on cell growth *in vitro* (Fig. 5A). The dynamics of tumor growth was monitored once a week over 5 weeks via *in vivo* bioluminescence imaging. We observed a delayed tumor growth for xenografts of chr11_BS79 and chr18_BS691 compared with the control, while chr1_BS1363 xenografts grew even faster than the control (Fig. 5B,C, Fig. S10). Tumor volumes of chr1_BS1363 and chr11_BS79 xenografts were significantly smaller as compared to control tumors (in case of chr11 one tumor did not grow at all). Xenografts of chr1_BS1363 were on average bigger than control tumors, although we observed considerable variability between individual tumors (Fig. 5D,E). These results confirm that targeting E‐boxes can decrease tumor growth *in vivo*. At the same time, they highlight the importance of *in vivo* validation, as additional factors may play a role and result in a different outcome than *in vitro*, as shown here in case of chr1_BS1363.

### 3.6 Implications of the sequence context on E‐box functionality

We observed that in some cases with a + 1 insertion, the inserted nucleotide did not change the E‐box motif (i.e., G added before the last G in the E‐box or C inserted after the first C in the E‐box). For some sgRNAs, the estimated frequency of such DNA edits reached up to ~ 50% (Table S12). Despite apparently not affecting the E‐box sequence, we did observe an effect on cell growth and expression of adjacent genes on bulk‐infected K562 cells. To gain further insights into the E‐box grammar, we focused on the E‐box CACATG on chromosome 10 (chr10_BS212) with a cut site directly before the last G in the E‐box (CACAT*G) and the highest percentage (56%) of +1 insertions with an additional G (CACGTGG).

**Fig. 6:**
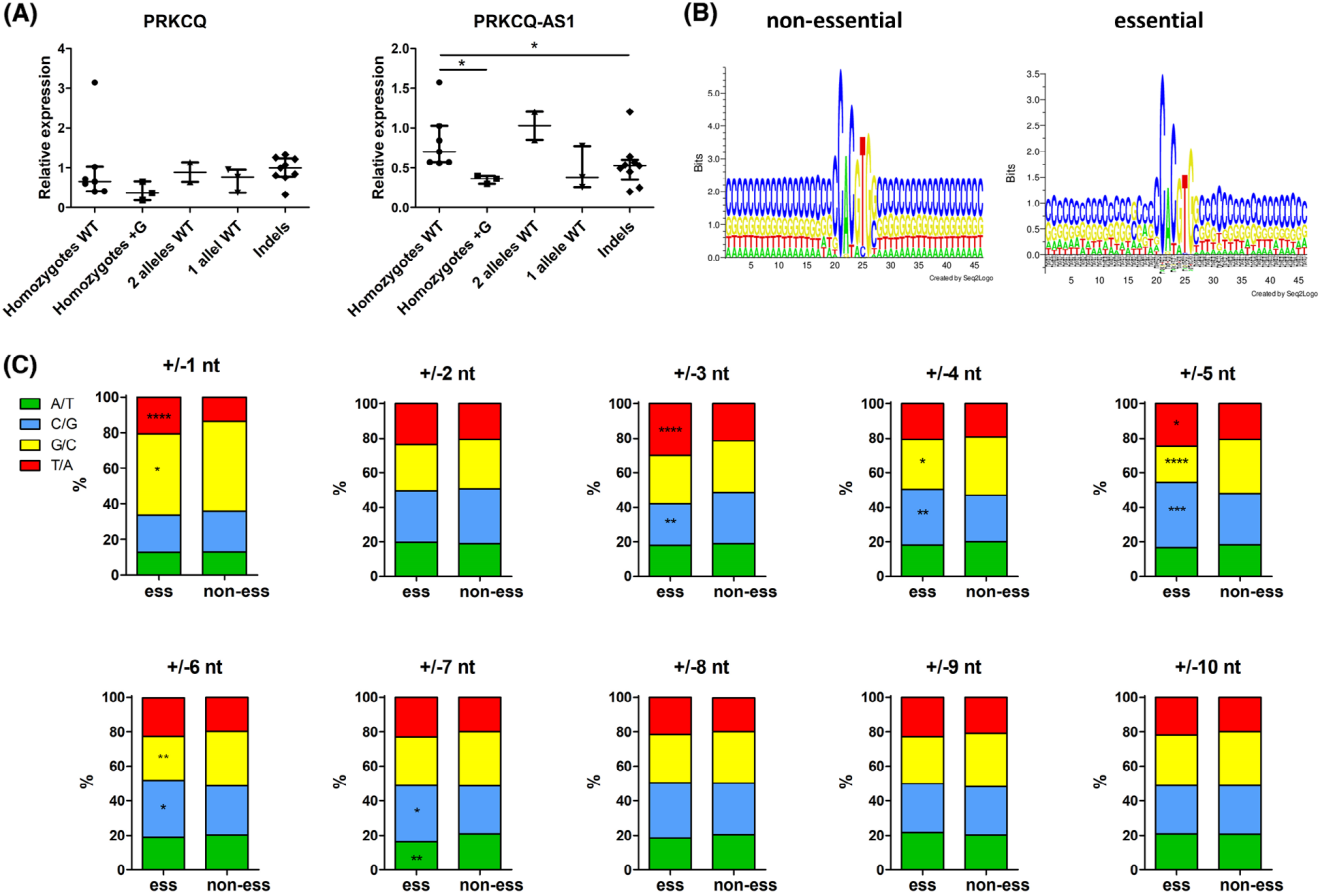
Grammar of the E‐box sequence context. (A) Expression of two genes adjacent to an E‐box on chromosome 10 (chr10_BS212) was examined in monoclonal cell lines derived from K562 cells transduced with an sgRNA targeting this E‐box. A spectrum of clones with various modifications of the E‐box was obtained, including wild‐type (WT) homozygotes (*n* = 7; K562 cells are triploid); homozygotes with the +G insertion after E‐box (*n* = 3); clones with two WT alleles and one mutated allele (*n* = 2); clones with one WT allele and two mutated alleles (*n* = 3); and clones with various indels on all three alleles (*n* = 9). Median with interquartile range is shown; *, *P* < 0.05, Kruskal–Wallis test with Dunn's post‐test. (B) Sequence logo (created using Seq2Logo) of the E‐boxes and 20 nt flanking sequences for non‐essential E‐boxes (left, *n* = 24 705) and E‐boxes essential in at least one cell line (right, *n* = 276). (C) Frequency of up to 10 nt upstream/downstream flanking essential (*n* = 276) and non‐essential (*n* = 24 705) E‐boxes. Since E‐boxes are (quasi)palindromic and can be read on either strand, G at +1 equals C at −1, etc. *, *P* < 0.05; **, *P* < 0.01; ***, *P* < 0.001; ****, *P* < 0.0001; chi‐squared goodness‐of‐fit test.

We successfully established 24 clones of K562 cells with varied mutations and/or WT sequence on all 3 alleles (K562 cells are triploid; Table S13). No significant differences between WT homozygotes and mutants were observed in the expression levels of the nearby PRKCQ gene that was not essential for K562 cells in the Brunello screen. By contrast, expression of the adjacent lncRNA PRKCQ‐AS1, whose downregulation negatively affected K562 cell growth, was significantly decreased in +G homozygotes, to a similar extent as in clones with other indels which clearly disrupted the E‐box (Fig. 6A). This indicated that even though the E‐box motif sequence *per se* was not changed, its functionality was affected.

This prompted us to look at the sequences flanking the E‐boxes. Analysis of the nucleotide frequency 20 nt upstream and downstream of non‐essential E‐boxes showed uniform distribution of nucleotides. By contrast, there were marked differences at particular positions flanking essential E‐boxes (Fig. 6B). Statistical analysis of 10 nt upstream/downstream revealed that certain nucleotides were significantly over‐ or underrepresented in the neighborhood of essential E‐boxes (Fig. 6C). Since E‐boxes are (quasi)palindromic and can be read on either strand, G at +1 equals C at −1, etc. The strongest bias in nucleotide composition was observed at positions ±1 nt, and 3–7 nt. In particular, we observed that G immediately after or C immediately before essential E‐boxes were unfavored. Thus, we speculate that the immediate context of E‐boxes might affect their functionality. This could explain why such changes without apparent disruption of the E‐box affected expression of adjacent genes and cell viability.

## 4 Discussion

Despite decades of research, MYC still evades full comprehension and therapeutic targeting. To understand the mechanisms underlying cancer cell dependency on MYC, it is essential to determine the crucial genes regulated by this TF. Therefore, the aim of this study was to identify on a genome‐wide scale functional MYC binding sites and corresponding target genes essential for cancer cell growth. To this end, we have established a novel CRISPR/Cas9‐based tool to disrupt MYC‐bound E‐boxes.

Extensive validation of our approach confirmed efficient E‐box disruption, decreased expression of adjacent genes and reduced MYC binding upon CRISPR/Cas9 editing of selected E‐boxes. No change in MYC binding for the E‐box on chr11 can be potentially explained by the presence of another, non‐essential E‐box, chr11_BS79_CACGCG ~100 bp downstream of the analyzed E‐box chr11_BS79_CGCGTG (Fig. S7B). Resolution of ChIP‐qPCR with DNA fragments of ca. 500 bp does not allow to distinguish such close binding sites. Finally, using individual sgRNAs we confirmed that E‐box disruption or knockout of adjacent genes significantly decreased K562 cell proliferation, in line with the results of the screens. However, we observed some discrepancies using a parallel dCas9 approach. We validated the effect on cell growth for two out of three E‐boxes, but for chr10_BS212, we did not observe decreased proliferation with dCas9, despite a similar effect on expression of two adjacent genes, *PRKCQ* and *PRKCQ‐AS1*. Similar inconsistencies have also been reported for targeting p53‐binding sites with WT Cas9 and dCas9 with an overall limited overlap [51], and this phenomenon requires further investigation.

Notably, using our strategy, it was possible to determine which E‐boxes are essential for cell viability and identify relevant regulated target genes. This is important since we demonstrated that not all E‐boxes in a binding site affected cell growth and target‐gene expression. Similarly, not all genes adjacent to an E‐box responded to E‐box disruption and were crucial for cancer cells. In our screen, 68–80% of essential E‐boxes were not localized near essential genes, but this might be an underestimation as we checked only the first TSS within 50 kb up‐ and downstream of each E‐box. Moreover, due to chromatin organization, the relevant target may be much further away. In addition, MYC also regulates noncoding RNAs, which were not included in this general analysis, yet might be essential. Combination of the MYC‐EBOX‐CRISPR screen with single‐cell RNA sequencing would add another layer of information to this experimental setup and allow direct identification of genes responding to E‐box disruption.

Essential genes identified in the Brunello screen showed a substantial overlap, and 77% of them belong to the panel of pan‐essential genes [52]. The number of essential genes identified in MCF7 was lower than in other cell lines (354 vs. 1226–1992). This may be due to less efficient gene knockout as these cells are hypertriploid to hypotetraploid. Previous genome‐wide screens in MCF7 cells also revealed a highly variable number of essential genes (527–2463) [53]. GO and GSEA analyses revealed processes common to all studied cell lines, which indicates that different types of cancers rely on the same factors for their growth [54]. On the contrary, the overlap between essential E‐boxes was very limited and analysis of adjacent genes revealed a bigger spectrum of cell‐type‐specific processes. This observation is in line with the fact that MYC acts within the predefined transcriptional landscape, which varies between cell types and developmental stages, and regulates expression of specific target genes [17, 21, 55, 56].

A recent study identified 1344 MYC‐dependent genes (log2FC < −0.58, *P* < 0.05) in K562 cells using SLAM‐seq upon MYC disruption [57]. 1035 of these genes had an E‐box within 50 kb that was included in the MYC‐EBOX‐CRISPR library. Of these, 284 genes were localized near an E‐box that was at least 1.5‐fold depleted in our screen in K562 cells (Fig. S11). This limited overlap may be due to the fact that in our study we focused on MYC targets which were essential for K562 cell growth, while SLAM‐seq included all targets. On the contrary, due to sgRNA design in our screen we might have missed some E‐boxes relevant for target genes. This highlights the need for integration of multiple approaches for identification of essential MYC targets.

Interestingly, our study provided also some novel insights into the grammar of E‐boxes and their surrounding sequences. We observed significantly different frequencies of specific nucleotides at certain positions in essential vs. nonessential E‐boxes. This observation provides novel indications about E‐box functionality and demonstrates the usefulness of high‐throughput CRISPR/Cas9 mutagenesis for studying TF‐binding sites. Previous studies showed that DNA flexibility and structure determined by the flanking sequences impact binding of TFs [58], including closely related TFs from the bHLH family: MYC, MAX, and MAD [59, 60]. Phylogenetic comparisons revealed strong sequence conservation of E‐boxes and also their flanking regions among species [61]. Moreover, it was recently reported that MYC is first engaged in open chromatin regions via non‐specific binding, while recognition of specific sequences stabilizes binding of MYC to DNA and promotes its transcriptional activity [62].

A limitation of our study is that we were not able to target all E‐boxes within the MYC‐bound loci. Our library was designed for the canonical E‐box motif (CACGTG), and two most common non‐canonical ones (CATGTG and CACGCG) [18, 20, 63, 64]. 29 811 (51%) of the 58 503 MYC peaks from ChIP data contained at least one of those E‐boxes. Other, less common E‐box motifs not included in our design (CACGAG, CACGAT, CATGCG, CACGTT, and CACGCT) together contributed only to 6348 sites (11%), while the remaining MYC peaks did not contain any of the above‐mentioned E‐boxes. It has been observed that MYC can also bind to regions without any known E‐box sequence [20, 63], but those binding sites cannot be targeted with our approach. For the 43 153 E‐boxes identified within the 29 811 MYC peaks, we were able to design sgRNAs targeting 24 981 E‐boxes. This was due to either lack of PAM sequence nearby (35% of E‐boxes not included in our library) or strong off‐target activity of designed sgRNAs (only 4% of E‐boxes not targeted). This could be overcome with a complementary approach using variant Cas9 nucleases with different PAM requirements.

A potential flaw in our approach could be the fact that other bHLH proteins can bind to E‐boxes and affect transcription [65]. Therefore, effect of E‐box disruption might be also related to other interactors. However, several findings strongly suggest that MYC is involved: (a) we focused on validated MYC binding sites (based on available MYC‐ChIP‐seq data); (b) luciferase reporter assay for selected E‐boxes showed decreased transcription after MYC knockdown; (c) ChIP confirmed that E‐box disruption reduced MYC binding. Altogether, this indicates that the activity of the studied E‐boxes is at least in part regulated by MYC, although we cannot exclude involvement of other factors.

## 5 Conclusions

In summary, the combined high‐throughput screens using the MYC‐EBOX‐CRISPR library targeting E‐boxes and the Brunello library for gene knockout is a useful tool for genome‐wide identification of E‐boxes which are important for MYC‐dependent networks in cancer cells. This well‐validated novel approach allows for the identification of essential MYC‐bound E‐boxes and the regulated target genes in MYC‐dependent cancers. The broad design enables studies in a variety of cancer cell types and determination of common as well as cell‐type‐specific targets. Further testing in normal cells may facilitate identification of potential novel therapeutic targets.

## Conflict of interest

The authors declare no conflict of interest.

## Author contributions

AD‐K planned and supervised the project and acquired funding. MK, MŻ, MP, MEK, WS, WŁ, IZ‐S, and AD‐K performed the experiments. TW designed the MYC‐EBOX‐CRISPR library sgRNAs; MK, MP, WŁ, NR, and AD‐K analyzed the data. NR, JK, and AvdB contributed to project conceptualization. MK and AD‐K prepared the figures and wrote the manuscript. All authors read and approved the manuscript.

### Peer review

The peer review history for this article is available at https://www.webofscience.com/api/gateway/wos/peer‐review/10.1002/1878‐0261.13493.

## Supporting information


**File S1.** .bed file with coordinates of targeted E‐boxes from MYC‐EBOX‐CRISPR library.

**Fig. S1.** Studied cell lines express high levels of MYC and depend on MYC for their growth.
**Fig. S2.** Quality of MYC‐CRISPR and Brunello libraries based on NGS.
**Fig. S3.** Changes in sgRNA abundance in two screen replicates.
**Fig. S4.** Validation of the screen results using the CRISPR/dCas9 approach.
**Fig. S5.** Snapshots of common E‐boxes and target genes in cancer cell lines.
**Fig. S6.** Snapshot of selected specific E‐boxes and target genes in cancer cell lines.
**Fig. S7.** Genomic location of sgRNAs targeting selected E‐boxes.
**Fig. S8.** Validation of selected E‐boxes in K562 cells.
**Fig. S9.** Validation of selected E‐boxes in ST486 cells.
**Fig. S10.** Dynamics of tumor growth *in vivo*.
**Fig. S11.** Intersection of the MYC‐CRISPR screen with SLAM‐seq data.
**Table S1.** List of E‐boxes and targeting sgRNAs in the MYC‐EBOX‐CRISPR library.
**Table S2.** Primer sequences.
**Table S3.** Oligo sequences.
**Table S4.** Number of reads obtained by NGS for individual samples.
**Table S5.** Raw read counts for MYC‐EBOX‐CRISPR library in two screen replicates.
**Table S6.** Raw read counts for Brunello library in two screen replicates.
**Table S7.** Screen results for Brunello library by gene and by sgRNA.
**Table S8.** Screen results for MYC‐EBOX‐CRISPR library by E‐box and by sgRNA.
**Table S9.** Top 50 enriched gene sets among genes from Brunello library.
**Table S10.** Top 50 enriched gene sets among genes adjacent to essential E‐boxes.
**Table S11.** Depleted genes near essential E‐boxes specific for each cell line—processes they are involved in and TCGA data regarding expression in normal vs. tumor tissues and association with survival.
**Table S12.** E‐box editing with CRISPR/Cas9: +1 insertions.
**Table S13.** Mutations of K562 clones.

## Data Availability

The script used to generate the MYC‐EBOX‐CRISPR library is deposited on github: https://github.com/tomaszwozniakihg/cas9_search_tool. MYC‐EBOX‐CRISPR library was deposited in Addgene (#173195).
