# Supplementary material for: CRISPR/Cas9 screen for genome‐wide interrogation of essential MYC‐bound E‐boxes in cancer cells
Source: Mol Oncol. 2023 Aug 7;17(11):2295–313. doi: 10.1002/1878-0261.13493 (PMC10620128; doi:10.1002/1878-0261.13493)
Supplement: Supplementary file 2 — Fig. S1. Studied cell lines express high levels of MYC and depend on MYC for their growth. Fig. S2. Quality of MYC‐CRISPR and Brunello libraries based on NGS. Fig. S3. Changes in sgRNA abundance in two screen replicates. Fig. S4. Validation of the screen results using the CRISPR/dCas9 approach. Fig. S5. Snapshots of common E‐boxes and target genes in cancer cell lines. Fig. S6. Snapshot of selected specific E‐boxes and target genes in cancer cell lines. Fig. S7. Genomic location of sgRNAs targeting selected E‐boxes. Fig. S8. Validation of selected E‐boxes in K562 cells. Fig. S9. Validation of selected E‐boxes in ST486 cells. Fig. S10. Dynamics of tumor growth in vivo. Fig. S11. Intersection of the MYC‐CRISPR screen with SLAM‐seq data. Table S1. List of E‐boxes and targeting sgRNAs in the MYC‐EBOX‐CRISPR library. Table S2. Primer sequences. Table S3. Oligo sequences. Table S4. Number of reads obtained by NGS for individual samples. Table S5. Raw read counts for MYC‐EBOX‐CRISPR library in two screen replicates. Table S6. Raw read counts for Brunello library in two screen replicates. Table S7. Screen results for Brunello library by gene and by sgRNA. Table S8. Screen results for MYC‐EBOX‐CRISPR library by E‐box and by sgRNA. Table S9. Top 50 enriched gene sets among genes from Brunello library. Table S10. Top 50 enriched gene sets among genes adjacent to essential E‐boxes. Table S11. Depleted genes near essential E‐boxes specific for each cell line—processes they are involved in and TCGA data regarding expression in normal vs. tumor tissues and association with survival. Table S12. E‐box editing with CRISPR/Cas9: +1 insertions. Table S13. Mutations of K562 clones. [file MOL2-17-2295-s002.zip › mol213493-sup-0002-Supinfo.pdf]

A CRISPR/Cas9 screen for genome-wide interrogation of essential MYC-bound E-boxes in cancer cells

Marta Kazimierska[1,2], Marta Podralska[1], Magdalena Żurawek[1], Tomasz Woźniak[1], Marta Elżbieta Kasprzyk[1], Weronika Sura[1], Wojciech Łosiewski[1], Iwona Ziółkowska-Suchanek[1], Joost Kluiver[3], Anke van den Berg[3], Natalia Rozwadowska[1], Agnieszka Dzikiewicz-Krawczyk[1]*

[1]Institute of Human Genetics, Polish Academy of Sciences, Poznań, Poland

[2]Institute of Bioorganic Chemistry, Polish Academy of Sciences, Poznań, Poland

[3]Department of Pathology and Medical Biology, University of Groningen, University Medical Center Groningen, Groningen, The Netherlands

SUPPLEMENTARY TABLES

Table S2. Primer sequences.

Primer name	Sequence
**sgRNA LIBRARY AMPLIFICATION**	
**oligo-F**	GTAACTTGAAAGTATTTCGATTTCTTGGCTTTATATATCTTGTGGAAAGGACGAAACACC
**oligo-R**	ACTTTTTCAAGTTGATAACGGACTAGCCTTATTTTAACTTGCTATTTCTAGCTCTAAAAC
**NGS LIBRARY PREPARATION**	
**Fwd-1**	AATGATACGGCGACCACCGAGATCTACACTCTTTCCCTACACGACGCTCTTCCGATCTTAAGTAGAGGCTTTATATATCTTGTGGAAAGGACGAAACACC
**Fwd-2**	AATGATACGGCGACCACCGAGATCTACACTCTTTCCCTACACGACGCTCTTCCGATCTATCATGCTTAGCTTTATATATCTTGTGGAAAGGACGAAACACC
**Fwd-3**	AATGATACGGCGACCACCGAGATCTACACTCTTTCCCTACACGACGCTCTTCCGATCTGATGCACATCTGCTTTATATATCTTGTGGAAAGGACGAAACACC
**Fwd-4**	AATGATACGGCGACCACCGAGATCTACACTCTTTCCCTACACGACGCTCTTCCGATCTCGATTGCTCGACGCTTTATATATCTTGTGGAAAGGACGAAACACC
**Fwd-5**	AATGATACGGCGACCACCGAGATCTACACTCTTTCCCTACACGACGCTCTTCCGATCTTCGATAGCAATTCGCTTTATATATCTTGTGGAAAGGACGAAACACC
**Fwd-6**	AATGATACGGCGACCACCGAGATCTACACTCTTTCCCTACACGACGCTCTTCCGATCTATCGATAGTTGCTTGCTTTATATATCTTGTGGAAAGGACGAAACACC
**Fwd-7**	AATGATACGGCGACCACCGAGATCTACACTCTTTCCCTACACGACGCTCTTCCGATCTGATCGATCCAGTTAGGCTTTATATATCTTGTGGAAAGGACGAAACACC
**Fwd-8**	AATGATACGGCGACCACCGAGATCTACACTCTTTCCCTACACGACGCTCTTCCGATCTCGATCGATTTGAGCCTGCTTTATATATCTTGTGGAAAGGACGAAACACC
**Fwd-9**	AATGATACGGCGACCACCGAGATCTACACTCTTTCCCTACACGACGCTCTTCCGATCTACGATCGATACACGATCGCTTTATATATCTTGTGGAAAGGACGAAACACC
**Fwd-10**	AATGATACGGCGACCACCGAGATCTACACTCTTTCCCTACACGACGCTCTTCCGATCTTACGATCGATGGTCCAGAGCTTTATATATCTTGTGGAAAGGACGAAACACC
**Rev-1**	CAAGCAGAAGACGGCATACGAGATTCGCCTTGGTGACTGGAGTTCAGACGTGTGCTCTTCCGATCTCCGACTCGGTGCCACTTTTTCAA
**Rev-2**	CAAGCAGAAGACGGCATACGAGATATAGCGTCGTGACTGGAGTTCAGACGTGTGCTCTTCCGATCTCCGACTCGGTGCCACTTTTTCAA

Primer name	Sequence
**Rev-3**	CAAGCAGAAGACGGCATACGAGATGAAGAAGTGTGACTGGAGTTCAGACGTGTGCTCTTCCGATCTCCGACTCGGTGCCACTTTTTCAA
**Rev-4**	CAAGCAGAAGACGGCATACGAGATATTCTAGGGTGACTGGAGTTCAGACGTGTGCTCTTCCGATCTCCGACTCGGTGCCACTTTTTCAA
**Rev-5**	CAAGCAGAAGACGGCATACGAGATCGTTACCAGTGACTGGAGTTCAGACGTGTGCTCTTCCGATCTCCGACTCGGTGCCACTTTTTCAA
**Rev-6**	CAAGCAGAAGACGGCATACGAGATGTCTGATGGTGACTGGAGTTCAGACGTGTGCTCTTCCGATCTCCGACTCGGTGCCACTTTTTCAA
**Rev-7**	CAAGCAGAAGACGGCATACGAGATTTACGCACGTGACTGGAGTTCAGACGTGTGCTCTTCCGATCTCCGACTCGGTGCCACTTTTTCAA
**Rev-8**	CAAGCAGAAGACGGCATACGAGATTTGAATAGGTGACTGGAGTTCAGACGTGTGCTCTTCCGATCTCCGACTCGGTGCCACTTTTTCAA
**TIDE PRIMERS**	
**chr17_BS377_TIDE-F**	CCCTGATCTTGCCAAGCAGA
**chr17_BS377_TIDE-R**	CCCTCCCCAACTTTCAGGAC
**chr10_BS212_TIDE-F**	ACCCGGCACCTCTAGCCA
**chr10_BS212_TIDE-R**	CCCGCAAGGCATAAAAAGTCC
**chr11_BS79_TIDE-F**	AGTCACTTCCGGAACTGCTG
**chr11_BS79_TIDE-R**	CCCACGCTGTCCAAGATCTT
**chr19_BS2255_TIDE-F**	CCTCTCCCGAAGTCACGATG
**chr19_BS2255_TIDE-R**	CGGCGGGCATTTGGAAATAG
**chr13_BS121_TIDE-F**	CGGTTGCCTTTCTTTCGCAA
**chr13_BS121_TIDE-R**	AGCCGACGCCTGACTTTAAA
**chr11_BS2113_TIDE-F**	GCACACCGCTTTGTCATTGT
**chr11_BS2113_TIDE-R**	TCAGTTCCGCTTGAGGCATT
**chr2_BS1664_TIDE-F**	CCCTGGCTTCAGCAGAATCA
**chr2_BS1664_TIDE-R**	GGACACAGGACCAGCTTCTG
**chr3_BS897_TIDE-F**	AGGAAGAGCTTTCTGGTGGC
**chr3_BS897_TIDE-R**	GCTTGGACCCTTTCTCCTCC
**qPCR PRIMERS**	
**RPLP2-F**	GATCTTGGACAGCGTGGGTA
**RPLP2-R**	GCAATGACGTCTTCAATGTTTTT
**SNRPD2-F**	GAAACGGGAGTGAACGGAG
**SNRPD2-R**	TGGGGTCATCTCACTCTTGG
**QPCTL-F**	AACTGGATCCACAGCGTCTC
**QPCTL-R**	AAGAGCAGTTGCAGGGTCAC
**CTC1-F**	CAGCTGTCACCCACGTGTC
**CTC1-R**	TGGACACTCGCAGTTCTGTC
**PRKRIR-F**	CTTCCTGCCTTATGAAGCCGA
**PRKRIR-R**	CCTGGCCACGACAATACTCC
**PRKCQ-F**	CAAGTGCCACGAGTTCACTG
**PRKCQ-R**	GCACTGGTAGCCCTGTTTGT
**PFAS-F**	GTGAGTGGATCAAGCCCATC
**PFAS-R**	GTAGACGGGACCTCCAACCT
**PIDD1-F**	GCTTCCTCCAACCGGTCAC
**PIDD1-R**	GAGGGGCCCAGTACAACAG
**PRKCQ-AS1-F**	CCCACAACTCGGAACTTGGA
**PRKCQ-AS1-R**	AGGAAGGATGCAAGACGTGG
**LINC00412-F**	CGGGGTTGTCACTGGAAGTT
**LINC00412-R**	CAGCAAAGTTGTACCATGACCC
**RPL21-F**	CCTTTGGCCACATATATGCGAATC
**RPL21-R**	ACACTTGTGGGGCATTCCTT

Primer name	Sequence
**SCAP-F**	ATCTCGGGCCTTCTACAACC
**SCAP-R**	CAAGGGGAGTTTCAGCAGTG
**PTPN23-F**	GAGGGCATGAAGGTCTCCTG
**PTPN23-R**	GCATGAGGTTGACGTTGAGC
**TBP-F**	GCCCGAAACGCCGAATAT
**TBP-R**	CCGTGGTTCGTGGCTCTCT
**LUCIFERASE CONSTRUCTS**	
**chr10_BS212_luc-F**	ATATGTGAGCTCCGCTCGCCAGCCTCC
**chr10_BS212_luc-R**	ATATGTCTCGAGCAGATACAGGAAAAGTCCCAGGT
**chr11_BS79_luc-F**	TAACGAGAGCTCGGTGCTAGGTACCGAAGGC
**chr11_BS79_luc-R**	TAACGACTCGAGAAAAATACCCCGCCGCCC
**chr17_BS377_luc-F**	ATTCGTGAGCTCTGCTTGCTTTATGGCCTTAACTAAC
**chr17_BS377_luc-R**	ATTCGTCTCGAGTGAGGGCTGTATTCCATGACC
**ChIP-qPCR**	
**chr10_BS212_ChIP-F**	CCTCTCGCCCAACTGAAAAC
**chr10_BS212_ChIP-R**	CAGAGAAAAGAGACCCAGCTC
**chr11_BS79_ChIP-F**	GTCCCTTTGGACTCGCTTC
**chr11_BS79_ChIP-R**	GTTCCGGAAGTGACTGCTCT
**chr17_BS377_ChIP-F**	TTTCGTCATCTAGCCCAAGC
**chr17_BS377_ChIP-R**	GGGCACCAAGTAGACACAGC

Table S3. Oligo sequences.

Oligo name	Sequence
**sgRNA**	
**chr17_BS377_sg1-S**	CACCGACGCGGTAACTATACTCACG
**chr17_BS377_sg1-AS**	AAACCGTGAGTATAGTTACCGCGTC
**chr17_BS377_sg2-S**	CACCGGGAGGATCCAGGTCCGCACG
**chr17_BS377_sg2-AS**	AAACCGTGCGGACCTGGATCCTCCC
**chr17_BS377_sg3-S**	CACCGGTGAGTATAGTTACCGCGTG
**chr17_BS377_sg3-AS**	AAACCACGCGGTAACTATACTCACC
**chr11_BS2113_sg1-S**	CACCGAAGCCAGCAGGTGAGACATG
**chr11_BS2113_sg1-AS**	AAACCATGTCTCACCTGCTGGCTTC
**chr10_BS212_sg1-S**	CACCGTTGGGCGAGAGGGAGACATG
**chr10_BS212_sg1-AS**	AAACCATGTCTCCCTCTCGCCCAAC
**chr11_BS79_sg1-S**	CACCGCGGTCACCACACTCACGCGG
**chr11_BS79_sg1-AS**	AAACCCGCGTGAGTGTGGTGACCGC
**chr11_BS79_sg2-S**	CACCGGCCCGGTCACCACACTCACG
**chr11_BS79_sg2-AS**	AAACCGTGAGTGTGGTGACCGGGCC
**chr2_BS1664_sg1-S**	CACCGCAAGTAGTAGGCTCGGCACG
**chr2_BS1664_sg1-AS**	AAACCGTGCCGAGCCTACTACTTGC
**chr19_BS2255_sg1-S**	CACCGAGCGTAGTGACCATCATGTG
**chr19_BS2255_sg1-AS**	AAACCACATGATGGTCACTACGCTC
**chr19_BS2255_sg2-S**	CACCGCCTAGCCCGGCCTCACATGA
**chr19_BS2255_sg2-AS**	AAACTCATGTGAGGCCGGGCTAGGC
**chr13_BS121_sg1-S**	CACCGAGCGCCCGGGAGCCACGCGT
**chr13_BS121_sg1-AS**	AAACACGCGTGGCTCCCGGGCGCTC
**chr13_BS121_sg2-S**	CACCGTTCCAGGTAGGGCCTACGCG
**chr13_BS121_sg2-AS**	AAACCGCGTAGGCCCTACCTGGAAC
**chr3_BS897_sg1-S**	CACCGCGCAGCCGTGGCTCATGTGA
**chr3_BS897_sg1-AS**	AAACTCACATGAGCCACGGCTGCGC
**chr3_BS897_sg2-S**	CACCGTCGCAGCCGTGGCTCATGTG
**chr3_BS897_sg2-AS**	AAACCACATGAGCCACGGCTGCGAC
**chr1_BS1363_sg1-S**	CACCGGTTGGTTGGAGCGAGCATGT
**chr1_BS1363_sg1-AS**	AAACACATGCTCGCTCCAACCAACC
**chr18_BS691_sg1-S**	CACCGGTCCCGGCGCCGCGCCATGT
**chr18_BS691_sg1-AS**	AAACACATGGCGCGGCGCCGGGACC
**PFAS_sg1-S**	CACCGAAGCCCATCATGTTTAGTGG
**PFAS_sg1-AS**	AAACCCACTAAACATGATGGGCTTC
**PFAS_sg2-S**	CACCGTGGACCCAAAAGTCGCCGCC
**PFAS_sg2-AS**	AAACGGCGGCGACTTTTGGGTCCAC
**PRKCQ_sg1-S**	CACCGCGATGATGTTGAGTGCACGA
**PRKCQ_sg1-AS**	AAACTCGTGCACTCAACATCATCGC
**PRKCQ_sg2-S**	CACCGGCTCCATCAAAAATGAAGCA
**PRKCQ_sg2-AS**	AAACTGCTTCATTTTTGATGGAGCC
**RPLP2_sg1-S**	CACCGGGACAGCGTGGGTATCGAGG
**RPLP2_sg1-AS**	AAACCCTCGATACCCACGCTGTCCC
**RPLP2_sg2-S**	CACCGGGACGACGACCGGCTCAACA
**RPLP2_sg2-AS**	AAACTGTTGAGCCGGTCGTCGTCCC
**PIDD1_sg1-S**	CACCGAGGGCGTCATGAGGACCCAG
**PIDD1_sg1-AS**	AAACCTGGGTCCTCATGACGCCCTC
**PIDD1_sg2-S**	CACCGGGGGGCGTCTAGCAGCTCAG
**PIDD1_sg2-AS**	AAACCTGAGCTGCTAGACGCCCCCC
**shRNA**	
**NT2-S**	GATCCGCAACAAGATGAAGAGCACCAACTCTTCAAGAGAGTTGTTCTACTTCTCGTGGTTGAGTTTTTG
**NT2-AS**	GCGTTGTTCTACTTCTCGTGGTTGAGAAGTTCTCTCAACAAGATGAAGAGCACCAACTCAAAAACTTAA
**PRKCQ-AS1_sh1-S**	GATCCACGCTAGAAAGGGCTTGTAAATTCAAGAGATTTACAAGCCCTTTCTAGCGTTTTTTG
**PRKCQ-AS1_sh1-AS**	AATTCAAAAAACGCTAGAAAGGGCTTGTAAATCTCTTGAATTTACAAGCCCTTTCTAGCGTG
**PRKCQ-AS1_sh2-S**	GATCCGGGATTTAGGATAGAAATTAATTCAAGAGATTAATTTCTATCCTAAATCCCTTTTTG
**PRKCQ-AS1_sh2-AS**	AATTCAAAAAGGGATTTAGGATAGAAATTAATCTCTTGAATTAATTTCTATCCTAAATCCCG
**MYC_sh1-S**	GATCCGATGAGGAAGAAATCGATGTTCAAGAGACATCGATTTCTTCCTCATCTTTTTG
**MYC_sh1-AS**	AATTCAAAAAGATGAGGAAGAAATCGATGTCTCTTGAACATCGATTTCTTCCTCATCG

Oligo name	Sequence
**MYC_sh3-S**	GATCCAACGACGAGAACAGTTGAAACATTCAAGAGATGTTTCAACTGTTCTCGTCGTTTTTTG
**MYC_sh3-AS**	AATTCAAAAAAACGACGAGAACAGTTGAAACATCTCTTGAATGTTTCAACTGTTCTCGTCGTTG
**MYC responsive element**	
**MYC_RE_S**	CCACGTGCACGTGCACGTGCACGTGCACGTGCACGTGC
**MYC_RE_AS**	TCGAGCACGTGCACGTGCACGTGCACGTGCACGTGCACGTGGAGCT

Table S4. Number of reads obtained by NGS for individual samples.

NGS pool	Sample	# reads	# perfect sgRNA matches	Coverage
**1**	MYC-CRISPR library plasmid	116,452,060	106,981,131	2,308x
	Brunello library plasmid	157,895,429	144,707,272	1,868x
**2**	K562_MYC-CRISPR #1 T0	83,489,199	75,443,224	1,627x
	K562_MYC-CRISPR #1 T1	59,895,534	53,260,435	1,149x
	K562_MYC-CRISPR #2 T0	63,067,319	56,420,523	1,217x
	K562_MYC-CRISPR #2 T1	58,484,870	52,205,808	1,126x
**3**	K562_Brunello #1 T0	54,221,760	49,649,571	641x
	K562_Brunello #1 T1	65,892,957	58,862,286	760x
	K562_Brunello #2 T0	72,332,476	66,210,988	855x
	K562_Brunello #2 T1	66,241,094	59,146,212	764x
**4**	ST486_MYC-CRISPR #1 T0	79 321 637	72 156 055	1,556x
	ST486_MYC-CRISPR #1 T1	87 870 982	79 467 416	1,714x
	ST486_MYC-CRISPR #2 T0	59 934 111	54 499 953	1,175x
	ST486_MYC-CRISPR #2 T1	70 122 820	63 352 176	1,366x
**5**	ST486_Brunello #1 T0	70 116 091	63 554 293	820x
	ST486_Brunello #1 T1	89 895 834	80 185 600	1,035x
	ST486_Brunello #2 T0	70 283 191	63 729 565	823x
	ST486_Brunello #2 T1	75 121 180	67 041 436	865x
**6**	HepG2_MYC-CRISPR #1 T0	51,447,782	47,155,897	1,017x
	HepG2_MYC-CRISPR #1 T1	66,626,438	60,555,248	1,306x
	HepG2_MYC-CRISPR #2 T0	50,858,585	46,650,129	1,006x
	HepG2_MYC-CRISPR #2 T1	52,090,164	47,337,827	1,021x
**7**	HepG2_Brunello #1 T0	44,194,540	33,638,192	434x
	HepG2_Brunello #1 T1	54,746,834	49,087,636	634x
	HepG2_Brunello #2 T0	60,630,324	55,661,488	719x
	HepG2_Brunello #2 T1	52,464,546	47,052,280	608x
**8**	MCF7_MYC-CRISPR #1 T0	55,595,928	50,892,310	1,098x
	MCF7_MYC-CRISPR #1 T1	63,075,305	57,089,710	1,232x
	MCF7_MYC-CRISPR #2 T0	50,858,585	46,650,129	1,006x
	MCF7_MYC-CRISPR #2 T1	50,339,659	45,688,415	986x
**9**	MCF7_Brunello #1 T0	53,498,499	48,765,805	630x
	MCF7_Brunello #1 T1	68,312,092	61,109,079	789x
	MCF7_Brunello #2 T0	55,841,233	51,347,850	663x
	MCF7_Brunello #2 T1	45,446,430	37,428,965	483x

Table S9. Top 50 enriched gene sets among genes from Brunello library.

	K562			ST486			HepG2			MCF7		
PROCESSES	RANK	NES	FDR q-val	RANK	NES	FDR q-val	RANK	NES	FDR q-val	RANK	NES	FDR q-val
KEGG_RIBOSOME	1	-2.28	0.0000	3	-1.81	0.0000	1	-2.02	0.0000	8	-3.14	0.0000
REACTOME_EUKARYOTIC_TRANSLATION_ELONGATION	3	-2.24	0.0000	2	-1.82	0.0000	6	-1.98	0.0000	5	-3.16	0.0000
REACTOME_RRNA_PROCESSING	6	-2.19	0.0000	7	-1.80	0.0000	5	-1.98	0.0000	2	-3.29	0.0000
REACTOME_EUKARYOTIC_TRANSLATION_INITIATION	10	-2.17	0.0000	1	-1.82	0.0000	8	-1.97	0.0000	3	-3.22	0.0000
REACTOME_TRANSLATION	5	-2.20	0.0000	9	-1.80	0.0000	10	-1.94	0.0000	1	-3.33	0.0000
REACTOME_RESPONSE_OF_EIF2AK4_GCN2_TO_AMINO_ACID_DEFICIENCY	8	-2.19	0.0000	8	-1.80	0.0000	2	-1.99	0.0000	7	-3.14	0.0000
WP_CYTOPLASMIC_RIBOSOMAL_PROTEINS	2	-2.24	0.0000	11	-1.79	0.0000	4	-1.98	0.0000	9	-3.11	0.0000
REACTOME_NONSENSE_MEDIATED_DECAY_NMD	4	-2.22	0.0000	4	-1.80	0.0000	11	-1.94	0.0000	10	-3.10	0.0000
KEGG_SPLICEOSOME	11	-2.16	0.0000	5	-1.80	0.0000	7	-1.97	0.0000	17	-2.96	0.0000
REACTOME_MRNA_SPLICING	15	-2.13	0.0000	6	-1.80	0.0000	9	-1.95	0.0000	14	-3.06	0.0000
REACTOME_INFLUENZA_INFECTION	13	-2.13	0.0000	10	-1.79	0.0000	18	-1.92	0.0000	4	-3.17	0.0000
REACTOME_SELENOAMINO_ACID_METABOLISM	9	-2.19	0.0000	12	-1.78	0.0000	12	-1.94	0.0000	13	-3.07	0.0000
REACTOME_SRP_DEPENDENT_COTRANSLATIONAL_PROTEIN_TARGETING_TO_MEMBRANE	7	-2.19	0.0000	37	-1.74	0.0000	3	-1.99	0.0000	12	-3.07	0.0000
REACTOME_PROCESSING_OF_CAPPED_INTRON_CONTAINING_PRE_MRNA	17	-2.12	0.0000	21	-1.76	0.0000	15	-1.93	0.0000	6	-3.15	0.0000
REACTOME_REGULATION_OF_EXPRESSION_OF_SLITS_AND_ROBOS	12	-2.14	0.0000	23	-1.76	0.0000	21	-1.91	0.0000	11	-3.08	0.0000
REACTOME_ACTIVATION_OF_THE_MRNA_UPON_BINDING_OF_THE_CAP_BINDING_COMPLEX_AND_EIFS_AND_SUBSEQUENT_BINDING_TO_43S	20	-2.11	0.0000	14	-1.78	0.0000	13	-1.94	0.0000	21	-2.92	0.0000
WP_MRNA_PROCESSING	22	-2.10	0.0000	15	-1.77	0.0000	17	-1.92	0.0000	25	-2.86	0.0000
HALLMARK_MYC_TARGETS_V1	18	-2.12	0.0000	26	-1.76	0.0000	22	-1.91	0.0000	15	-2.98	0.0000
BILANGES_SERUM_AND_RAPAMYCIN_SENSITIVE_GENES	16	-2.12	0.0000	24	-1.76	0.0000	19	-1.92	0.0000	23	-2.86	0.0000
REACTOME_RRNA_MODIFICATION_IN_THE_NUCLEUS_AND_CYTOSOL	19	-2.11	0.0000	17	-1.76	0.0000	23	-1.91	0.0000	30	-2.83	0.0000
REACTOME_MRNA_SPLICING_MINOR_PATHWAY	26	-2.07	0.0000	13	-1.78	0.0000	20	-1.91	0.0000	31	-2.82	0.0000
REACTOME_CELLULAR_RESPONSE_TO_STARVATION	27	-2.07	0.0000	16	-1.77	0.0000	36	-1.89	0.0000	18	-2.96	0.0000
REACTOME_MITOCHONDRIAL_TRANSLATION	21	-2.11	0.0000	20	-1.76	0.0000	35	-1.89	0.0000	22	-2.87	0.0000
CHNG_MULTIPLE_MYELOMA_HYPERPLOID_UP	14	-2.13	0.0000	27	-1.76	0.0000	29	-1.90	0.0000	51	-2.68	0.0000
REACTOME_CHROMOSOME_MAINTENANCE	28	-2.06	0.0000	42	-1.73	0.0000	31	-1.90	0.0000	35	-2.79	0.0000
REACTOME_DNA_REPLICATION	39	-2.04	0.0000	41	-1.73	0.0000	39	-1.88	0.0000	20	-2.93	0.0000
REACTOME_TELOMERE_MAINTENANCE	29	-2.06	0.0000	47	-1.73	0.0000	27	-1.90	0.0000	42	-2.74	0.0000
REACTOME_SIGNALING_BY_ROBO_RECEPTORS	25	-2.07	0.0000	63	-1.71	0.0000	40	-1.88	0.0000	19	-2.95	0.0000
REACTOME_TRANSCRIPTION_COUPLED_NUCLEOTIDE_EXCISION_REPAIR_TC_NER	24	-2.08	0.0000	35	-1.74	0.0000	16	-1.92	0.0000	84	-2.57	0.0000
REACTOME_SNRNP_ASSEMBLY	46	-2.02	0.0000	30	-1.75	0.0000	48	-1.85	0.0000	46	-2.73	0.0000
TIEN_INTESTINE_PROBIOTICS_6HR_UP	23	-2.08	0.0000	62	-1.71	0.0000	32	-1.90	0.0000	63	-2.64	0.0000
REACTOME_DUAL_INCISION_IN_TC_NER	41	-2.03	0.0000	39	-1.74	0.0000	14	-1.94	0.0000	108	-2.51	0.0000
REACTOME_DNA_REPLICATION_PRE_INITIATION	66	-1.99	0.0000	72	-1.70	0.0001	41	-1.87	0.0000	36	-2.78	0.0000
REACTOME_DNA_STRAND_ELONGATION	38	-2.04	0.0000	31	-1.75	0.0000	24	-1.91	0.0000	124	-2.48	0.0000
WP_DNA_REPLICATION	42	-2.03	0.0000	60	-1.71	0.0000	28	-1.90	0.0000	89	-2.56	0.0000
REACTOME_HIV_TRANSCRIPTION_ELONGATION	34	-2.05	0.0000	18	-1.76	0.0000	26	-1.90	0.0000	148	-2.44	0.0000
REACTOME_EXTENSION_OF_TELOMERES	37	-2.04	0.0000	89	-1.69	0.0001	33	-1.89	0.0000	69	-2.62	0.0000
KEGG_DNA_REPLICATION	31	-2.05	0.0000	28	-1.76	0.0000	34	-1.89	0.0000	137	-2.46	0.0000
REACTOME_HDR_THROUGH_HOMOLOGOUS_RECOMBINATION_HRR	35	-2.05	0.0000	88	-1.69	0.0001	58	-1.84	0.0000	53	-2.67	0.0000
HALLMARK_MYC_TARGETS_V2	36	-2.05	0.0000	40	-1.73	0.0000	125	-1.80	0.0000	44	-2.74	0.0000
REACTOME_FORMATION_OF_RNA_POL_II_ELONGATION_COMPLEX	30	-2.05	0.0000	44	-1.73	0.0000	57	-1.85	0.0000	115	-2.49	0.0000
REACTOME_RNA_POLYMERASE_II_TRANSCRIBES_SNRNA_GENES	86	-1.97	0.0000	59	-1.71	0.0000	85	-1.82	0.0000	39	-2.78	0.0000
REACTOME_ORC1_REMOVAL_FROM_CHROMATIN	82	-1.98	0.0000	71	-1.70	0.0001	47	-1.85	0.0000	70	-2.62	0.0000
REACTOME_TRANSPORT_OF_MATURE_TRANSCRIPT_TO_CYTOPLASM	81	-1.98	0.0000	80	-1.70	0.0001	81	-1.82	0.0000	32	-2.81	0.0000
WONG_EMBRYONIC_STEM_CELL_CORE	61	-2.00	0.0000	127	-1.67	0.0002	64	-1.84	0.0000	27	-2.86	0.0000
REACTOME_NUCLEOTIDE_EXCISION_REPAIR	45	-2.02	0.0000	92	-1.69	0.0001	56	-1.85	0.0000	90	-2.55	0.0000
REACTOME_HIV_LIFE_CYCLE	55	-2.00	0.0000	110	-1.69	0.0001	78	-1.83	0.0000	47	-2.72	0.0000
WP_TRANSLATION_FACTORS	125	-1.93	0.0000	22	-1.76	0.0000	53	-1.85	0.0000	96	-2.54	0.0000
DANG_MYC_TARGETS_UP	40	-2.04	0.0000	161	-1.66	0.0003	62	-1.84	0.0000	34	-2.79	0.0000
MANALO_HYPOXIA_DN	59	-2.00	0.0000	138	-1.67	0.0002	82	-1.82	0.0000	26	-2.86	0.0000
REACTOME_S_PHASE	73	-1.98	0.0000	105	-1.69	0.0001	93	-1.82	0.0000	37	-2.78	0.0000
REACTOME_TRANSCRIPTION_OF_THE_HIV_GENOME	33	-2.05	0.0000	45	-1.73	0.0000	69	-1.83	0.0000	162	-2.41	0.0000
REACTOME_HIV_INFECTION	98	-1.95	0.0000	82	-1.70	0.0001	112	-1.80	0.0000	33	-2.81	0.0000

	K562			ST486			HepG2			MCF7		
**PROCESSES**	**RANK**	**NES**	**FDR q-val**	**RANK**	**NES**	**FDR q-val**	**RANK**	**NES**	**FDR q-val**	**RANK**	**NES**	**FDR q-val**
REACTOME_FORMATION_OF_TC_NER_PRE_INCISION_COMPLEX	72	-1.98	0.0000	43	-1.73	0.0000	25	-1.91	0.0000	190	-2.38	0.0000
REACTOME_HOMOLOGY_DIRECTED_REPAIR	50	-2.01	0.0000	166	-1.66	0.0003	95	-1.81	0.0000	40	-2.77	0.0000
REACTOME_G2_M_CHECKPOINTS	75	-1.98	0.0000	128	-1.67	0.0002	127	-1.79	0.0000	29	-2.85	0.0000
REACTOME_TELOMERE_C_STRAND_LAGGING_STRAND_SYNTHESIS	70	-1.99	0.0000	108	-1.69	0.0001	38	-1.89	0.0000	144	-2.45	0.0000
REACTOME_ACTIVATION_OF_ATR_IN_RESPONSE_TO_REPLICATION_STRESS	47	-2.02	0.0000	106	-1.69	0.0001	80	-1.83	0.0000	132	-2.46	0.0000
REACTOME_CELL_CYCLE_CHECKPOINTS	106	-1.94	0.0000	126	-1.67	0.0002	118	-1.80	0.0000	16	-2.97	0.0000
REACTOME_FORMATION_OF_THE_EARLY_ELONGATION_COMPLEX	56	-2.00	0.0000	19	-1.76	0.0000	30	-1.90	0.0000	269	-2.25	0.0000
IRITANI_MAD1_TARGETS_DN	89	-1.96	0.0000	38	-1.74	0.0000	128	-1.79	0.0000	125	-2.47	0.0000
WP_EUKARYOTIC_TRANSCRIPTION_INITIATION	51	-2.01	0.0000	34	-1.74	0.0000	84	-1.82	0.0000	216	-2.33	0.0000
REN_BOUND_BY_E2F	32	-2.05	0.0000	184	-1.64	0.0004	50	-1.85	0.0000	135	-2.46	0.0000
CROONQUIST_NRAS_SIGNALING_DN	48	-2.02	0.0000	197	-1.64	0.0006	65	-1.84	0.0000	113	-2.50	0.0000
REACTOME_TRANSPORT_OF_MATURE_MRNAS_DERIVED_FROM_INTRONLESS_TRANSCRIPTS	111	-1.94	0.0000	70	-1.70	0.0001	207	-1.75	0.0001	48	-2.70	0.0000
KEGG_RNA_POLYMERASE	84	-1.97	0.0000	129	-1.67	0.0002	37	-1.89	0.0000	203	-2.35	0.0000
REACTOME_SEPARATION_OF_SISTER_CHROMATIDS	196	-1.88	0.0000	104	-1.69	0.0001	147	-1.78	0.0000	28	-2.85	0.0000
PID_ATR_PATHWAY	179	-1.89	0.0000	49	-1.72	0.0000	120	-1.80	0.0000	140	-2.45	0.0000
MODY_HIPPOCAMPUS_PRENATAL	44	-2.02	0.0000	250	-1.62	0.001	132	-1.79	0.0000	75	-2.61	0.0000
REACTOME_DNA_DOUBLE_STRAND_BREAK_REPAIR	68	-1.99	0.0000	201	-1.64	0.0006	185	-1.76	0.0001	49	-2.70	0.0000
REACTOME_PROCESSING_OF_CAPPED_INTRONLESS_PRE_MRNA	49	-2.01	0.0000	155	-1.66	0.0003	71	-1.83	0.0000	243	-2.29	0.0000
HALLMARK_E2F_TARGETS	93	-1.96	0.0000	217	-1.63	0.0007	172	-1.77	0.0001	45	-2.74	0.0000
REACTOME_HOST_INTERACTIONS_OF_HIV_FACTORS	200	-1.88	0.0000	121	-1.68	0.0001	171	-1.77	0.0001	38	-2.78	0.0000
REACTOME_POSTMITOTIC_NUCLEAR_PORE_COMPLEX_NPC_REFORMATION	228	-1.86	0.0000	36	-1.74	0.0000	176	-1.77	0.0001	104	-2.52	0.0000
REACTOME_MITOCHONDRIAL_TRNA_AMINOACYLATION	137	-1.92	0.0000	32	-1.74	0.0000	102	-1.81	0.0000	292	-2.22	0.0000
REACTOME_MITOTIC_METAPHASE_AND_ANAPHASE	233	-1.86	0.0000	151	-1.66	0.0002	156	-1.78	0.0000	24	-2.86	0.0000
REACTOME_TRNA_AMINOACYLATION	104	-1.95	0.0000	29	-1.75	0.0000	157	-1.78	0.0000	291	-2.22	0.0000
REACTOME_MITOTIC_SPINDLE_CHECKPOINT	220	-1.87	0.0000	143	-1.67	0.0002	184	-1.76	0.0001	41	-2.74	0.0000
KEGG_AMINOACYL_TRNA_BIOSYNTHESIS	69	-1.99	0.0000	33	-1.74	0.0000	224	-1.74	0.0002	263	-2.27	0.0000
REACTOME_CELL_CYCLE_MITOTIC	198	-1.88	0.0000	189	-1.64	0.0005	200	-1.75	0.0001	43	-2.74	0.0000
REACTOME_M_PHASE	254	-1.85	0.0000	190	-1.64	0.0005	219	-1.74	0.0001	50	-2.70	0.0000
REACTOME_REGULATION_OF_TP53_ACTIVITY_THROUGH_PHOSPHORYLATION	43	-2.02	0.0000	171	-1.65	0.0003	309	-1.70	0.0006	198	-2.36	0.0000
KEGG_OXIDATIVE_PHOSPHORYLATION	150	-1.91	0.0000	367	-1.58	0.003	42	-1.87	0.0000	255	-2.28	0.0000
REACTOME_RESPIRATORY_ELECTRON_TRANSPORT	114	-1.93	0.0000	561	-1.51	0.0146	45	-1.86	0.0000	122	-2.48	0.0000
REACTOME_RNA_POLYMERASE_I_TRANSCRIPTION_TERMINATION	103	-1.95	0.0000	46	-1.73	0.0000	76	-1.83	0.0000	640	-1.84	0.0052
XU_RESPONSE_TO_TRETINOIN_AND_NSC682994_DN	299	-1.82	0.0001	25	-1.76	0.0000	202	-1.75	0.0001	342	-2.17	0.0000
REACTOME_MICRORNA_MIRNA_BIOGENESIS	408	-1.75	0.0007	160	-1.66	0.0003	46	-1.85	0.0000	326	-2.19	0.0000
MOOTHA_VOXPHOS	262	-1.84	0.0001	493	-1.54	0.0082	44	-1.86	0.0000	217	-2.32	0.0000
WP_ELECTRON_TRANSPORT_CHAIN_OXPHOS_SYSTEM_IN_MITOCHONDRIA	232	-1.86	0.0000	505	-1.53	0.0092	43	-1.87	0.0000	395	-2.11	0.0001
REACTOME_NUCLEOBASE_BIOSYNTHESIS	453	-1.73	0.0012	50	-1.72	0.0000	528	-1.60	0.0072	654	-1.82	0.0065
WP_MITOCHONDRIAL_COMPLEX_I_ASSEMBLY_MODEL_OXPHOS_SYSTEM	263	-1.84	0.0001	1359	-1.32	0.1732	49	-1.85	0.0000	293	-2.22	0.0000
WANG_ADIPOGENIC_GENES_REPRESSED_BY_SIRT1	629	-1.65	0.0067	48	-1.72	0.0000	814	-1.50	0.0369	792	-1.71	0.0211

Table S10. Top 50 enriched gene sets among genes adjacent to essential E-boxes.

PROCESSES	K562			ST486			HepG2			MCF7		
	RANK	NES	FDR q-val	RANK	NES	FDR q-val	RANK	NES	FDR q-val	RANK	NES	FDR q-val
REACTOME_TRANSLATION	2	-1.72	0.003	19	-1.41	0.2655	17	-1.54	0.0324	3	-2.12	0.000
REACTOME_METABOLISM_OF_RNA	6	-1.67	0.0065	8	-1.45	0.2156	19	-1.52	0.0451	2	-2.24	0.000
REACTOME_RRNA_PROCESSING	3	-1.71	0.002	35	-1.37	0.2988	18	-1.53	0.0372	1	-2.25	0.000
REACTOME_SIGNALING_BY_ROBO_RECEPTORS	13	-1.64	0.0084	34	-1.37	0.307	6	-1.57	0.021	6	-2.04	0.000
REACTOME_INFLUENZA_INFECTION	8	-1.66	0.0086	31	-1.37	0.3028	16	-1.55	0.0212	11	-1.95	0.0012
HSIAO_HOUSEKEEPING_GENES	27	-1.58	0.034	17	-1.42	0.2092	12	-1.55	0.0238	10	-1.95	0.0012
REACTOME_REGULATION_OF_EXPRESSION_OF_SLITS_AND_ROBOS	21	-1.60	0.0205	37	-1.37	0.2875	2	-1.59	0.0285	7	-2.00	0.0005
REACTOME_CELLULAR_RESPONSE_TO_STARVATION	11	-1.64	0.0096	39	-1.37	0.2793	1	-1.59	0.0551	23	-1.88	0.0032
REACTOME_EUKARYOTIC_TRANSLATION_INITIATION	19	-1.61	0.0177	42	-1.36	0.2629	9	-1.56	0.0229	4	-2.09	0.000
WP_CYTOPLASMIC_RIBOSOMAL_PROTEINS	5	-1.67	0.0078	52	-1.34	0.3208	3	-1.59	0.0241	17	-1.92	0.002
KEGG_RIBOSOME	12	-1.64	0.0091	49	-1.34	0.3305	10	-1.56	0.0209	9	-1.96	0.0012
REACTOME_RESPONSE_OF_EIF2AK4_GCN2_TO_AMINO_ACID_DEFICIENCY	15	-1.64	0.0077	50	-1.34	0.327	4	-1.58	0.0199	13	-1.94	0.0014
REACTOME_EUKARYOTIC_TRANSLATION_ELONGATION	18	-1.62	0.0129	45	-1.35	0.322	7	-1.57	0.0192	12	-1.95	0.0014
REACTOME_NONSENSE_MEDIATED_DECAY_NMD	22	-1.60	0.0196	43	-1.36	0.2843	13	-1.55	0.025	5	-2.07	0.000
CAIRO_HEPATOBLASTOMA_CLASSES_UP	10	-1.65	0.0092	11	-1.44	0.1928	45	-1.41	0.2831	20	-1.90	0.0025
REACTOME_SELENOAMINO_ACID_METABOLISM	4	-1.68	0.0091	59	-1.33	0.3106	14	-1.55	0.0237	18	-1.92	0.0021
BERENJENO_TRANSFORMED_BY_RHOA_UP	24	-1.60	0.0203	15	-1.43	0.2146	36	-1.43	0.2413	21	-1.89	0.0028
WANG_TUMOR_INVASIVENESS_UP	26	-1.58	0.0303	7	-1.45	0.2385	29	-1.44	0.2351	34	-1.79	0.0174
REACTOME_SRP_DEPENDENT_COTRANSLATIONAL_PROTEIN_TARGETING_TO_MEMBRANE	14	-1.64	0.0083	48	-1.34	0.3371	20	-1.52	0.0492	15	-1.94	0.0014
DODD_NASOPHARYNGEAL_CARCINOMA_DN	30	-1.56	0.0571	9	-1.45	0.1996	39	-1.41	0.2917	28	-1.84	0.0073
REACTOME_METABOLISM_OF_AMINO_ACIDS_AND_DERIVATIVES	17	-1.63	0.0114	41	-1.36	0.2686	8	-1.57	0.0212	45	-1.71	0.0587
LEE_BMP2_TARGETS_DN	9	-1.66	0.0081	12	-1.44	0.214	69	-1.35	0.4071	24	-1.85	0.0063
HALLMARK_MYC_TARGETS_V1	28	-1.57	0.0424	3	-1.50	0.114	58	-1.38	0.3373	41	-1.72	0.0506
REACTOME_NERVOUS_SYSTEM_DEVELOPMENT	49	-1.50	0.1514	68	-1.32	0.3286	5	-1.58	0.0222	14	-1.94	0.0014
WONG_EMBRYONIC_STEM_CELL_CORE	7	-1.66	0.0078	98	-1.29	0.3406	25	-1.45	0.2048	8	-1.96	0.0014
REACTOME_INFECTIOUS_DISEASE	16	-1.63	0.0105	53	-1.34	0.319	11	-1.56	0.0224	61	0.00	0.000
REACTOME_CELLULAR_RESPONSES_TO_EXTERNAL_STIMULI	31	-1.56	0.0561	58	-1.33	0.313	15	-1.55	0.0223	49	-1.70	0.0677
PUJANA_BRCA1_PCC_NETWORK	34	-1.55	0.0564	27	-1.38	0.2877	54	-1.39	0.2867	57	0.00	0.000
TIEN_INTESTINE_PROBIOTICS_24HR_DN	38	-1.51	0.1435	94	-1.29	0.3332	21	-1.51	0.0656	90	0.00	0.000
PUJANA_CHEK2_PCC_NETWORK	32	-1.55	0.0575	115	-1.27	0.3674	70	-1.35	0.4194	31	-1.82	0.0113
LI_AMPLIFIED_IN_LUNG_CANCER	127	-1.39	0.3328	23	-1.39	0.3066	47	-1.40	0.2771	62	0.00	0.000
JISON_SICKLE_CELL_DISEASE_DN	46	-1.51	0.1329	174	-1.21	0.4354	31	-1.44	0.2311	16	-1.94	0.0014
REACTOME_DEVELOPMENTAL_BIOLOGY	117	-1.40	0.3227	70	-1.32	0.3318	46	-1.40	0.2797	38	-1.75	0.0362
OSMAN_BLADDER_CANCER_DN	1	-1.72	0.006	108	-1.27	0.3717	122	-1.28	0.5499	42	-1.72	0.0509
FISCHER_DREAM_TARGETS	39	-1.51	0.1448	56	-1.33	0.3169	171	-1.24	0.5691	26	-1.84	0.0077
DUTERTRE_ESTRADIOL_RESPONSE_6HR_UP	132	-1.39	0.3351	75	-1.31	0.3322	35	-1.43	0.2319	58	0.00	0.000
MANALO_HYPOXIA_DN	86	-1.44	0.2568	102	-1.28	0.3462	118	-1.28	0.553	19	-1.92	0.0021
KARLSSON_TGFB1_TARGETS_UP	111	-1.41	0.3038	107	-1.27	0.365	104	-1.29	0.5435	22	-1.89	0.0028
GARY_CD5_TARGETS_DN	79	-1.45	0.2469	202	-1.19	0.4502	38	-1.42	0.2825	30	-1.82	0.0104
KINSEY_TARGETS_OF_EWSR1_FLII_FUSION_UP	81	-1.44	0.2586	29	-1.38	0.3112	232	-1.19	0.6121	40	-1.73	0.0507
RODRIGUES_THYROID_CARCINOMA_POORLY_DIFFERENTIATED_UP	66	-1.46	0.2218	104	-1.28	0.3575	48	-1.40	0.2714	227	0.00	0.000
FOURNIER_ACINAR_DEVELOPMENT_LATE_2	183	-1.34	0.4391	21	-1.39	0.3041	128	-1.27	0.5785	119	0.00	0.000
MONNIER_POSTRADIATION_TUMOR_ESCAPE_UP	35	-1.54	0.0741	135	-1.24	0.4069	215	-1.20	0.6066	74	0.00	0.000
SENGUPTA_NASOPHARYNGEAL_CARCINOMA_UP	230	-1.30	0.5218	14	-1.43	0.2069	75	-1.34	0.4164	176	0.00	0.000
MARTENS_TRETINOIN_RESPONSE_DN	25	-1.59	0.025	238	-1.15	0.5105	195	-1.22	0.5705	43	-1.72	0.053
KIM_ALL_DISORDERS_OLIGODENDROCYTE_NUMBER_CORR_UP	47	-1.50	0.1457	22	-1.39	0.2978	63	-1.37	0.3542	371	0.00	0.000
REACTOME_PROCESSING_OF_CAPPED_INTRON_CONTAINING_PRE_MRNA	283	-1.24	0.654	110	-1.27	0.3682	78	-1.34	0.4316	32	-1.80	0.0162
MOOTHA_HUMAN_MITODB_6_2002	41	-1.51	0.1413	118	-1.26	0.3832	220	-1.19	0.6066	125	0.00	0.000
KEGG_PURINE_METABOLISM	107	-1.41	0.3088	66	-1.33	0.3064	290	-1.13	0.7009	44	-1.72	0.0531
REACTOME_MRNA_SPLICING	351	-1.18	0.742	186	-1.20	0.437	34	-1.43	0.2328	36	-1.77	0.0248
YAGI_AML_WITH_INV_16_TRANSLOCATION	29	-1.56	0.0506	164	-1.22	0.4216	196	-1.22	0.5746	266	0.00	0.000
MUELLER_PLURINET	56	-1.48	0.1869	362	-0.96	0.8146	209	-1.20	0.5963	33	-1.79	0.0176
DESERT_STEM_CELL_HEPATOCELLULAR_CARCINOMA_SUBCLASS_UP	136	-1.39	0.3375	16	-1.43	0.2056	126	-1.27	0.5787	418	0.00	0.000
REACTOME_CHROMATIN_MODIFYING_ENZYMES	33	-1.55	0.0563	341	-1.01	0.7537	149	-1.26	0.5413	211	0.00	0.000
BASAKI_YBX1_TARGETS_UP	238	-1.28	0.5525	13	-1.43	0.2172	168	-1.24	0.5479	332	0.00	0.000

	K562			ST486			HepG2			MCF7		
PROCESSES	RANK	NES	FDR q-val	RANK	NES	FDR q-val	RANK	NES	FDR q-val	RANK	NES	FDR q-val
IVANOVA_HEMATOPOIESIS_EARLY_PROGENITOR	45	-1.51	0.1329	103	-1.28	0.3531	210	-1.20	0.5972	394	0.00	0.000
GRYDER_PAX3FOXO1_TOP_ENHANCERS	385	-1.16	0.7788	4	-1.47	0.2276	270	-1.14	0.6886	100	0.00	0.000
LASTOWSKA_NEUROBLASTOMA_COPY_NUMBER_UP	42	-1.51	0.1412	145	-1.24	0.4113	272	-1.14	0.6907	300	0.00	0.000
BENPORATH_MYC_MAX_TARGETS	503	-1.04	0.926	20	-1.40	0.2568	108	-1.29	0.5543	197	0.00	0.000
WANG_RESPONSE_TO_GSK3_INHIBITOR_SB216763_DN	370	-1.17	0.7582	44	-1.35	0.2915	83	-1.33	0.4607	347	0.00	0.000
MARTINEZ_RB1_AND_TP53_TARGETS_DN	355	-1.18	0.7414	36	-1.37	0.2946	217	-1.20	0.6084	254	0.00	0.000
RHEIN_ALL_GLUCOCORTICOID_THERAPY_DN	23	-1.60	0.0193	156	-1.23	0.4058	94	-1.31	0.4875	606	0.00	0.000
KIM_BIPOLAR_DISORDER_OLIGODENDROCYTE_DENSITY_CORR_UP	65	-1.46	0.2154	133	-1.25	0.405	23	-1.46	0.2064	708	0.00	0.000
BLALOCK_ALZHEIMERS_DISEASE_DN	167	-1.36	0.3934	71	-1.31	0.3299	28	-1.44	0.2328	691	0.00	0.000
TARTE_PLASMA_CELL_VS_PLASMABLAST_UP	161	-1.36	0.3886	28	-1.38	0.2841	457	-0.95	0.8906	319	0.00	0.000
BUYTAERT_PHOTODYNAMIC_THERAPY_STRESS_DN	431	-1.12	0.8382	40	-1.36	0.2746	164	-1.24	0.5449	338	0.00	0.000
STARK_PREFRONTAL_CORTEX_22Q11_DELETION_DN	20	-1.61	0.0178	158	-1.23	0.41	43	-1.41	0.2884	790	0.00	0.000
MARTINEZ_TP53_TARGETS_DN	334	-1.19	0.7369	10	-1.44	0.2066	251	-1.16	0.6544	452	0.00	0.000
GRYDER_PAX3FOXO1_ENHANCERS_IN_TADS	569	-0.99	0.9607	38	-1.37	0.2822	346	-1.06	0.7977	123	0.00	0.000
REACTOME_CYTOKINE_SIGNALING_IN_IMMUNE_SYSTEM	321	-1.21	0.7069	33	-1.37	0.3046	487	-0.89	0.9443	419	0.00	0.000
BOUDOUKHA_BOUND_BY_IGF2BP2	43	-1.51	0.1384	219	-1.17	0.4797	123	-1.27	0.5558	1049	0.00	0.000
BAELDE_DIABETIC_NEPHROPATHY_DN	489	-1.05	0.9311	245	-1.15	0.5166	40	-1.41	0.2965	677	0.00	0.000
PHONG_TNF_RESPONSE_VIA_P38_COMPLETE	97	-1.43	0.2725	46	-1.35	0.3159	288	-1.13	0.7026	1148	0.00	0.000
LIU_SOX4_TARGETS_DN	556	-1.01	0.9379	5	-1.47	0.2098	389	-1.01	0.8655	712	0.00	0.000
ACEVEDO_NORMAL_TISSUE_ADJACENT_TO_LIVER_TUMOR_DN	513	-1.03	0.9373	18	-1.41	0.2512	205	-1.21	0.5789	1036	0.00	0.000
BOQUEST_STEM_CELL_CULTURED_VS_FRESH_UP	395	-1.15	0.7783	26	-1.38	0.2956	462	-0.94	0.893	1082	0.00	0.000
REACTOME_SIGNALING_BY_WNT	673	-0.87	1.0000	467	-0.59	1.0000	41	-1.41	0.299	1032	0.00	0.000
BLANCO_MELO_INFLUENZA_A_INFECTION_A594_CELLS_DN	780	-0.77	1.0000	436	-0.75	0.9705	30	-1.44	0.2306	1165	0.00	0.000
ALONSO_METASTASIS_UP				348	-0.99	0.779				35	-1.78	0.0201
BILANGES_SERUM_AND_RAPAMYCIN_SENSITIVE_GENES							22	-1.46	0.2011	50	-1.70	0.0673
BOYAULT_LIVER_CANCER_SUBCLASS_G3_UP	75	-1.45	0.248				26	-1.45	0.2072	498	0.00	0.000
BRUECKNER_TARGETS_OF_MIRLET7A3_UP										39	-1.73	0.045
CHICAS_RB1_TARGETS_GROWING	226	-1.30	0.5105				24	-1.46	0.2031	85	0.00	0.000
DANG_MYC_TARGETS_UP	141	-1.38	0.361	24	-1.39	0.3107				47	-1.71	0.0607
GAVIN_FOXP3_TARGETS_CLUSTER_T7	36	-1.54	0.082							95	0.00	0.000
GRAESSMANN_APOPTOSIS_BY_SERUM_DEPRIVATION_UP	723	-0.83	1.0000				37	-1.43	0.244	236	0.00	0.000
HOLLEMAN_VINCRISTINE_RESISTANCE_B_ALL_DN				30	-1.37	0.3098				305	0.00	0.000
HOLLMANN_APOPTOSIS_VIA_CD40_DN	592	-0.97	0.967				50	-1.40	0.289	694	0.00	0.000
LEE_EARLY_T_LYMPHOCYTE_UP	40	-1.51	0.1446							896	0.00	0.000
MALONEY_RESPONSE_TO_17AAG_DN	346	-1.19	0.7482	47	-1.34	0.3323				536	0.00	0.000
MARTINEZ_RESPONSE_TO_TRABECTEDIN_DN	119	-1.40	0.3242							27	-1.84	0.0075
MARZEC_IL2_SIGNALING_UP	37	-1.54	0.0817							543	0.00	0.000
NIKOLSKY_BREAST_CANCER_8Q23_Q24_AMPLICON				389	-0.91	0.8701	49	-1.40	0.277	1364	0.00	0.000
REACTOME_ACTIVATION_OF_THE_MRNA_UPON_BINDING_OF_THE_CAP_BINDING_COMPLEX_AND_EIFS_AND_SUBSEQUENT_BINDING_TO_43S										37	-1.76	0.0317
REACTOME_ASPARAGINE_N_LINKED_GLYCOSYLATION	609	-0.95	0.976				27	-1.45	0.2117	823	0.00	0.000
REACTOME_DISEASES_OF_GLYCOSYLATION	48	-1.50	0.1504	1	-1.52	0.1389				924	0.00	0.000
REACTOME_DISEASES_OF_METABOLISM	126	-1.39	0.3324	2	-1.51	0.1202				577	0.00	0.000
REACTOME_HIV_INFECTION	206	-1.32	0.4834				53	-1.39	0.2903	46	-1.71	0.0585
REACTOME_INTERFERON_SIGNALING	449	-1.09	0.8777	32	-1.37	0.3095				439	0.00	0.000
REACTOME_MITOCHONDRIAL_TRANSLATION	98	-1.43	0.27							48	-1.70	0.063
REACTOME_RRNA_MODIFICATION_IN_THE_NUCLEUS_AND_CYTOSOL	95	-1.43	0.2705				194	-1.22	0.5636	29	-1.83	0.0081
REACTOME_SIGNALING_BY_RECEPTOR_TYROSINE_KINASES	696	-0.85	1.0000	6	-1.46	0.2421				928	0.00	0.000
REACTOME_TCF_DEPENDENT_SIGNALING_IN_RESPONSE_TO_WNT	638	-0.91	1.0000				44	-1.41	0.2828	457	0.00	0.000
SCHLOSSER_SERUM_RESPONSE_DN	433	-1.11	0.8412				42	-1.41	0.2949	837	0.00	0.000
TIEN_INTESTINE_PROBIOTICS_6HR_UP	44	-1.51	0.1353							25	-1.85	0.0072
WANG_PROSTATE_CANCER_ANDROGEN_INDEPENDENT				25	-1.39	0.3019				261	0.00	0.000
WP_16P112_PROXIMAL_DELETION_SYNDROME	50	-1.50	0.1502							709	0.00	0.000
WP_PATHWAYS_AFFECTED_IN_ADENOID_CYSTIC_CARCINOMA							32	-1.44	0.2343			
YAGI_AML_SURVIVAL				265	-1.13	0.5477	33	-1.43	0.238	654	0.00	0.000

Table S11. Depleted genes near essential E-boxes specific for each cell line – processes they are involved in and TCGA data regarding expression in normal vs tumor tissues and association with survival.

Gene	Gene Ontology Biological Process	P value survival	Median expression normal tissue	Median expression tumor tissue	P value expression
**K562**					
CTU2	tRNA processing	N/A	N/A	N/A	N/A
INTS3	DNA repair	N/A	N/A	N/A	N/A
NAT10	rRNA processing, tRNA processing	N/A	N/A	N/A	N/A
NOP9	rRNA processing, ribosomal small subunit export from nucleus	N/A	N/A	N/A	N/A
PELP1	rRNA processing	N/A	N/A	N/A	N/A
MAP2K3	protein phosphorylation, MAPK cascade	N/A	N/A	N/A	N/A
MCRS1	DNA repair, chromatin organization, histone H4 acetylation	N/A	N/A	N/A	N/A
MRPL12	mitochondrial transcription and translation	N/A	N/A	N/A	N/A
MED16	transcription by RNA polymerase II	N/A	N/A	N/A	N/A
TMEM106A	positive regulation of MAPK cascade, innate immune response	N/A	N/A	N/A	N/A
SEC61A1	protein transport	N/A	N/A	N/A	N/A
**ST486**					
CDT1	DNA replication, cell cycle, chromosome organization	N/A	N/A	N/A	N/A
TRIM28	DNA-templated transcription, DNA repair, chromatin organization	N/A	N/A	N/A	N/A
TFRC	positive regulation of B cell proliferation, signal transduction	N/A	N/A	N/A	N/A
NUP62	mRNA transport, protein transport, signal transduction	N/A	N/A	N/A	N/A
UBE2G2	protein ubiquitination	N/A	N/A	N/A	N/A
SNX8	protein transport	N/A	N/A	N/A	N/A
OGDH	generation of precursor metabolites and energy	N/A	N/A	N/A	N/A
CHMP2A	nucleus organization, protein transport, viral release from host cell	N/A	N/A	N/A	N/A
MCL1	programmed cell death	N/A	N/A	N/A	N/A
FUS	transcription by RNA polymerase II, DNA-templated transcription, RNA splicing	N/A	N/A	N/A	N/A
POLR3E	DNA-templated transcription, innate immune response	N/A	N/A	N/A	N/A
RPL22	translation	N/A	N/A	N/A	N/A
POLR2L	DNA-templated transcription, transcription by RNA polymerase I, transcription by RNA polymerase II	N/A	N/A	N/A	N/A
RPL12	translation	N/A	N/A	N/A	N/A
**HepG2**					
POLE4	DNA-templated DNA replication	0.00059	34.97	45.46	3.07E-12
SLX4	DNA repair, DNA replication, telomere maintenance	0.0024	0.333	0.999	1.62E-12
PIGT	attachment of GPI anchor to protein, neuron differentiation	0.0083	33.78	80.97	1.62E-12
MAEA	cell cycle, cytoskeleton organization, erythrocyte maturation	0.00056	13.44	22.38	1.62E-12
CTBP1	DNA-templated transcription, transcription by RNA polymerase II, histone acetylation	0.16	45.59	71.39	1.00E-12
GFPT1	protein N-linked glycosylation, carbohydrate derivative metabolic process	0.11	6.27	9.81	1.00E-12
POLR3E	DNA-templated transcription, innate immune response	0.021	6.77	11.06	1.62E-12
PLA2G10	phospholipid metabolic process	0.03	0	0	
RPLP0	ribosome biogenesis, translation, cellular response to interleukin-4	0.029	508.54	1014.39	1.00E-12
ASNS	asparagine biosynthetic process, apoptotic process, mitotic cell cycle	0.00028	0.74	2.6	1.62E-12
RPL18	translation	0.097	680.19	1163.4	1.62E-12
WDR43	rRNA processing, ribosome biogenesis, transcription elongation by RNA polymerase II, transcription by RNA polymerase I	0.00087	6.25	8.95	1.05E-11
MAD2L1BP	regulation of exit from mitosis	0.1	12.75	21.32	1.62E-12
PDE4DIP	centrosome cycle	0.93	24.6	29.3	4.66E-11

Gene	Gene Ontology Biological Process	P value survival	Median expression normal tissue	Median expression tumor tissue	P value expression
BFAR	protein polyubiquitination, apoptotic process	0.054	13	20.22	1.62E-12
TIMM9	protein transport	0.00011	13.54	27.78	1.62E-12
ATP5H	proton motive force-driven ATP synthesis	0.0066	266.18	457.47	1.00E-12
GCN1L1	translation, cellular response to amino acid starvation	0.0029	5.34	11.57	1.00E-12
**MCF7**					
DCLRE1B	DNA repair, telomere maintenance	0.76	6.07	6.45	1.43E-10
PHF2	chromatin organization, transcription by RNA polymerase II, histone lysine demethylation	0.28	29.75	24.06	6.08E-10
POLR3C	immune system process, DNA-templated transcription, transcription by RNA polymerase III	0.63	29.41	32.19	1.62E-12
PRIM1	DNA replication	0.39	8.46	11.8	1.00E-12
NCOA3	DNA-templated transcription, histone acetylation, stem cell division	0.095	17.05	16.08	3.16E-04
ZMYND8	DNA-templated transcription	0.0014	23.26	31.15	1.00E-12
ZNF217	DNA-templated transcription, transcription by RNA polymerase II	0.16	31.16	35.82	1.62E-12
GALK1	carbohydrate metabolic process, phosphorylation	0.71	11.7	18.38	1.62E-12
PARD6B	cell cycle, regulation of cell migration, axonogenesis	0.77	3.73	8.37	1.00E-12
RSBN1	chromatin organization	0.51	10.36	7.52	2.25E-10
BCAS1	myelination	0.52	3.36	8.78	1.62E-12
ANXA9	cell-cell adhesion, synaptic transmission	0.95	7.73	26.67	1.62E-12
DHX40	none	0.24	49.13	46.81	1.88E-05
SPATA2L	none	0.65	6.12	7.91	5.59E-08
C17orf64	none	0.81	0.11	0.025	4.46E-01
HEATR6	none	0.8	10.85	13.13	1.62E-12
LSM12	none	0.84	34.86	47.37	1.62E-12

Table S12. E-box editing with CRISPR/Cas9: +1 insertions.

sgRNA	E-box	% +1 insertions	% +G/C	% E-box not changed	Most prevalent E-box after +1 insertion
**Chr17_BS377_sg1**	CACGTG	9	20	1,8	CAACGTG
**Chr17_BS377_sg2**	CGCGTG	65	78	50	CGCGTGG
**Chr17_BS377_sg3**	CGCGTG	45	0	0 (cut in the middle)	-
**Chr11_BS2113_sg1**	CACATG	60	80	48	CACATGG
**Chr10_BS212_sg1**	CACATG	80	70	56	CACATGG
**Chr3_BS897_sg1**	CATGTG	0	0	0	-
**Chr3_BS897_sg2**	CATGTG	35	0	0 (cut in the middle)	-
**Chr11_BS79_sg1**	CGCGTG	9	40	3	CGCGTGG
**Chr11_BS79_sg2**	CGCGTG	17	0	0 (cut in the middle)	-
**Chr2_BS1664_sg1**	CACGTG	30	70	28	CACGTGG
**Chr19_BS2255_sg1**	CACATG	35	0	0 (cut in the middle)	-
**Chr19_BS2255_sg2**	CACATG	60	0	0 (cut in the middle)	-
**Chr13_BS121_sg1**	CGCGTG	0	0	0	-
**Chr13_BS121_sg2**	CGCGTG	55	62	34	CCGCGTG

Table S13. Mutations of K562 clones.

Name	Mutations
**B4**	wild type homozygous
**F10**	wild type homozygous
**C10**	wild type homozygous
**D6A**	wild type homozygous
**D6B**	wild type homozygous
**F6**	wild type homozygous
**G9**	wild type homozygous
**B2**	homozygous +G
**G4A**	homozygous +G
**G6A**	homozygous +G
**F9**	2 wild type alleles, deletion -17 ntt
**F11**	2 wild type alleles, deletion -39 nt
**D11**	1 wild type allele, deletions -37 nt and -54 nt
**B3**	1 wild type allele, 2x deletion -49 nt
**G3**	1 wild type allele, 2x deletion -29 nt
**G4B**	2x deletion -17 nt and insertion +G
**G7**	2x deletion -18 nt and insertion +G
**C8A**	deletions -15 nt and 2x -31 nt
**D2**	deletions -20 nt -19 nt and -4 nt
**D7**	Deletions -18 nt and 2x -15 nt
**D8**	2x deletion -41nt and +1 insertion
**B9**	deletions -43 nt and 2x -32 nt
**C9**	3x deletion >-200 nt
**G8**	3x deletion >-200 nt

SUPPLEMENTARY FIGURES

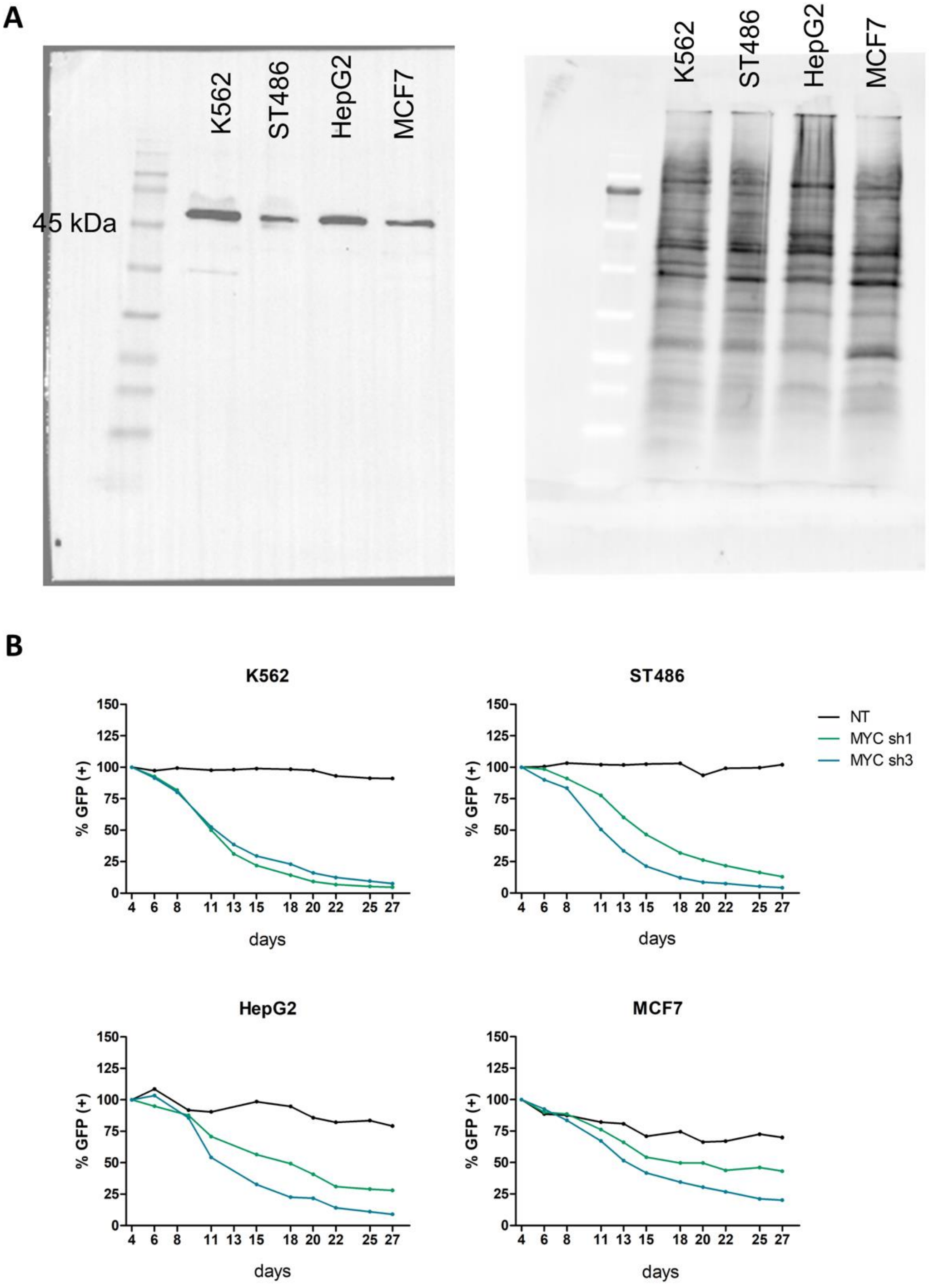

Figure S1. Studied cell lines express high levels of MYC and depend on MYC for their growth. A) Western blot with anti-MYC antibody (Abcam, Cambridge, UK, ab56, 1:1000) – left blot. Right blot presents total protein loading. **B)** GFP competition growth assay. Cells were transduced with shRNAs targeting MYC or a non-targeting control construct to achieve ~40-60% transduction efficiency. % of GFP+ (transduced) cells was measured by flow cytometry three times a week for 27 days and normalized to the first measurement on day 4.

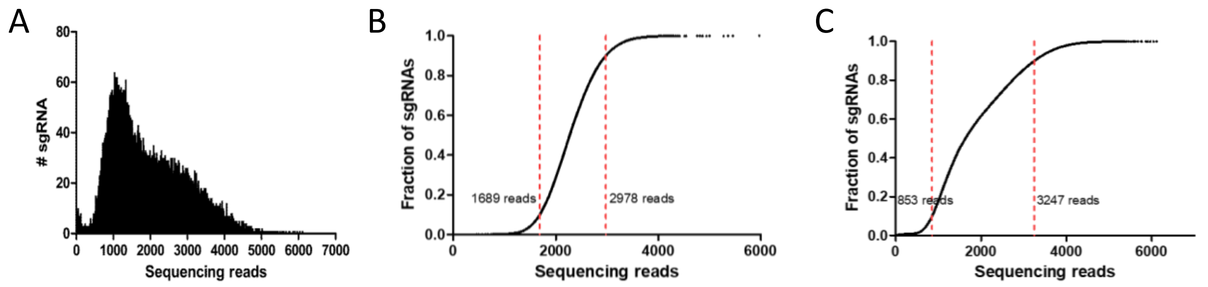

Figure S2. Quality of MYC-CRISPR and Brunello libraries based on NGS. A) Distribution of sgRNA read counts in the libraries. **B)** Cumulative frequency of sgRNAs. Red lines indicate the 10th and 90th percentile in MYC-CRISPR **C)** and Brunello library.

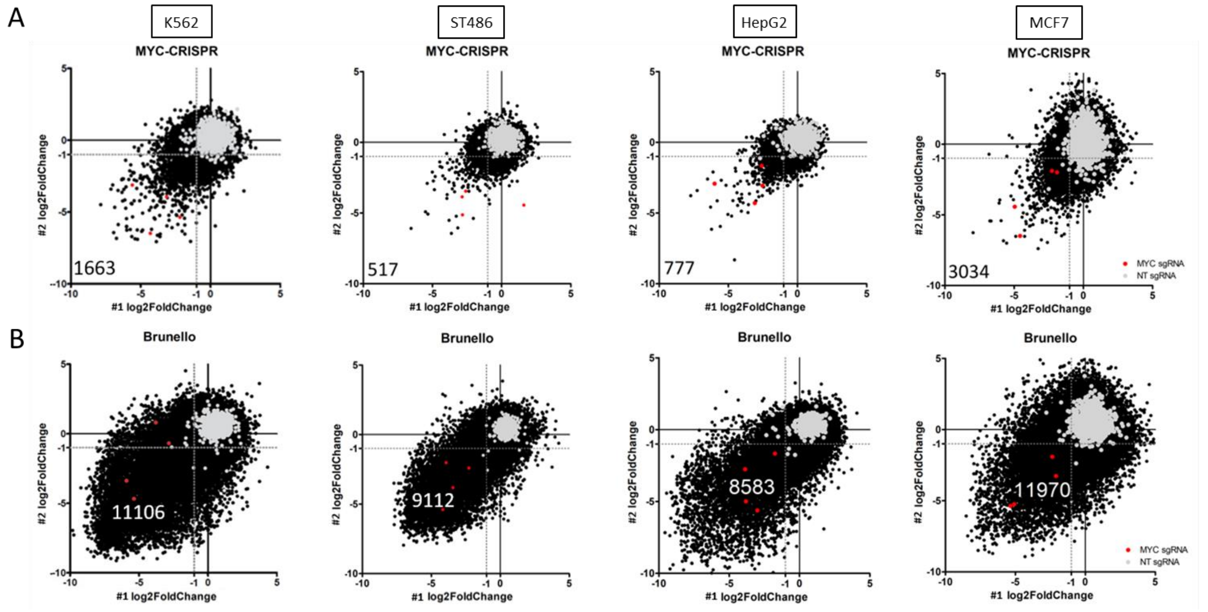

Figure S3. Changes in sgRNA abundance in two screen replicates with A) MYC-CRISPR and **B)** Brunello library. Log2 fold change values for replicate #1 and #2 are shown on the X and Y axis.

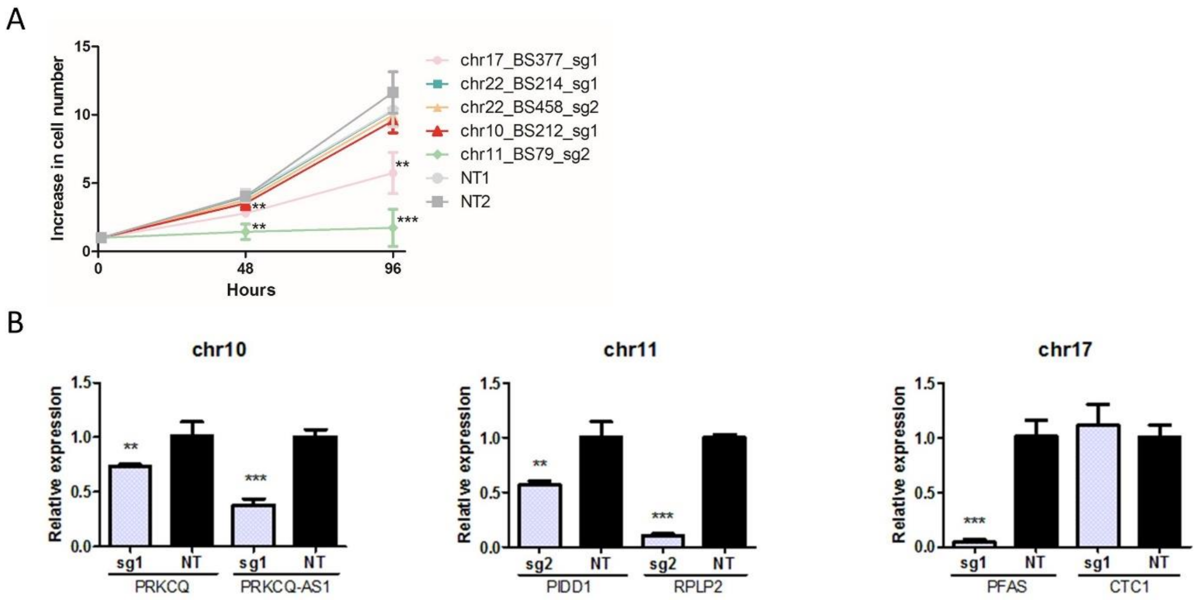

Figure S4. Validation of the screen results using the CRISPR/dCas9 approach. A) Cell viability after CRISPR/dCas9 blocking of selected E-boxes. Cells expressing dCas9 were infected with individual sgRNAs targeting E-boxes. After puromycin selection for four days, cell viability was measured using CellTiter-Glo assay at three timepoints: 0, 48 and 96 hours. Shown are average values and standard deviations from three independent experiments, each performed in triplicate. **, p<0.01; ***, p<0.001, Student's t-test **B)** qRT-PCR analysis of expression of genes adjacent to selected E-boxes upon CRISPR/dCas9 blocking of E-box sequences. For all E-boxes at least one nearby gene showed significantly decreased expression. The mean and SD of two independent experiments, each performed in triplicate, are shown. **, p<0.01; ***, p<0.001, Student's t-test.

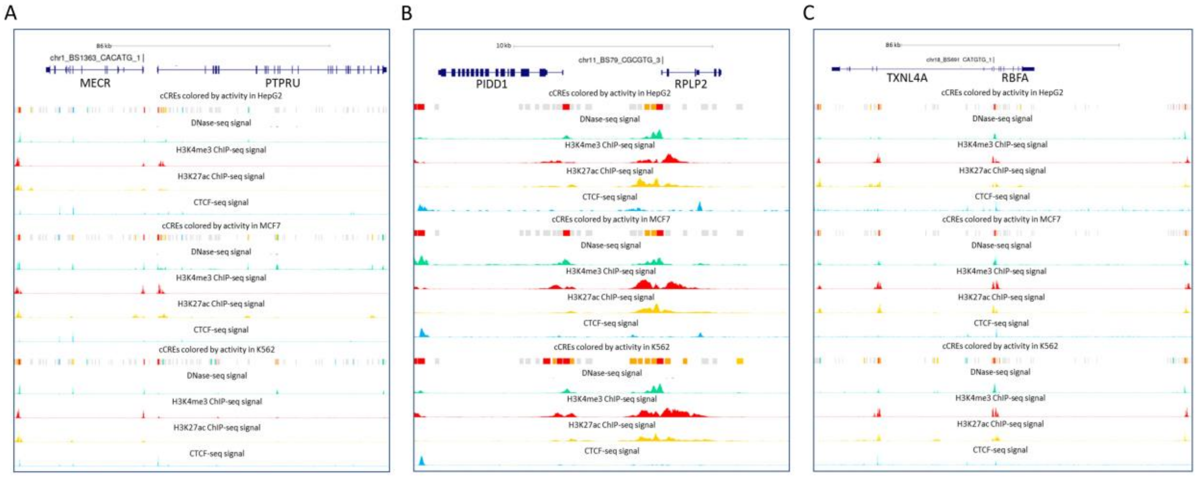

Figure S5. Snapshots of common E-boxes and target genes in cancer cell lines. A) chr1_BS1363 **B)** chr11_BS79 and **C)** chr18_BS691. Figure was prepared using SCREEN (https://screen.wenglab.org).

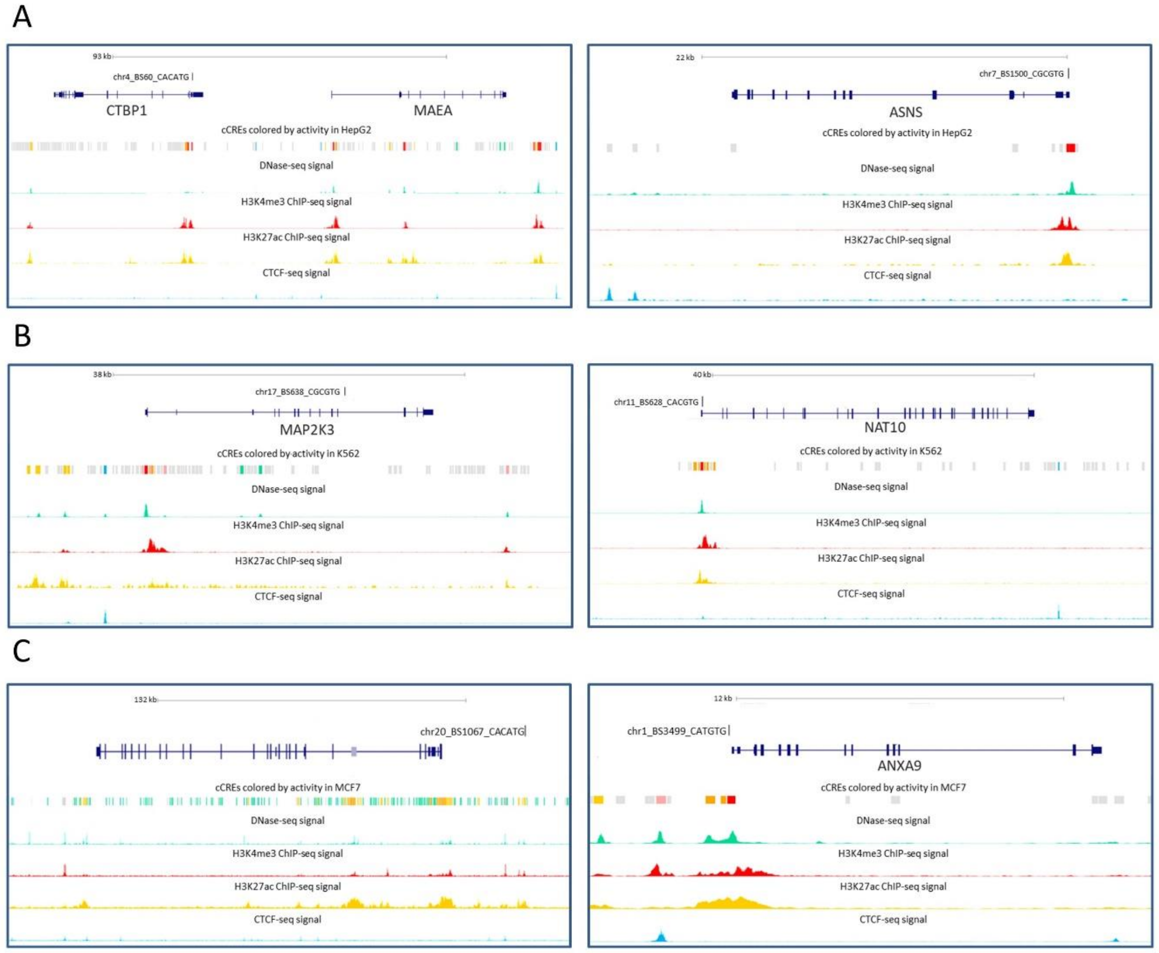

Figure S6. Snapshot of selected specific E-boxes and target genes in cancer cell lines. A) HepG2**, B)** K562 and **C)** MCF7**.** Figure was prepared using SCREEN (https://screen.wenglab.org).

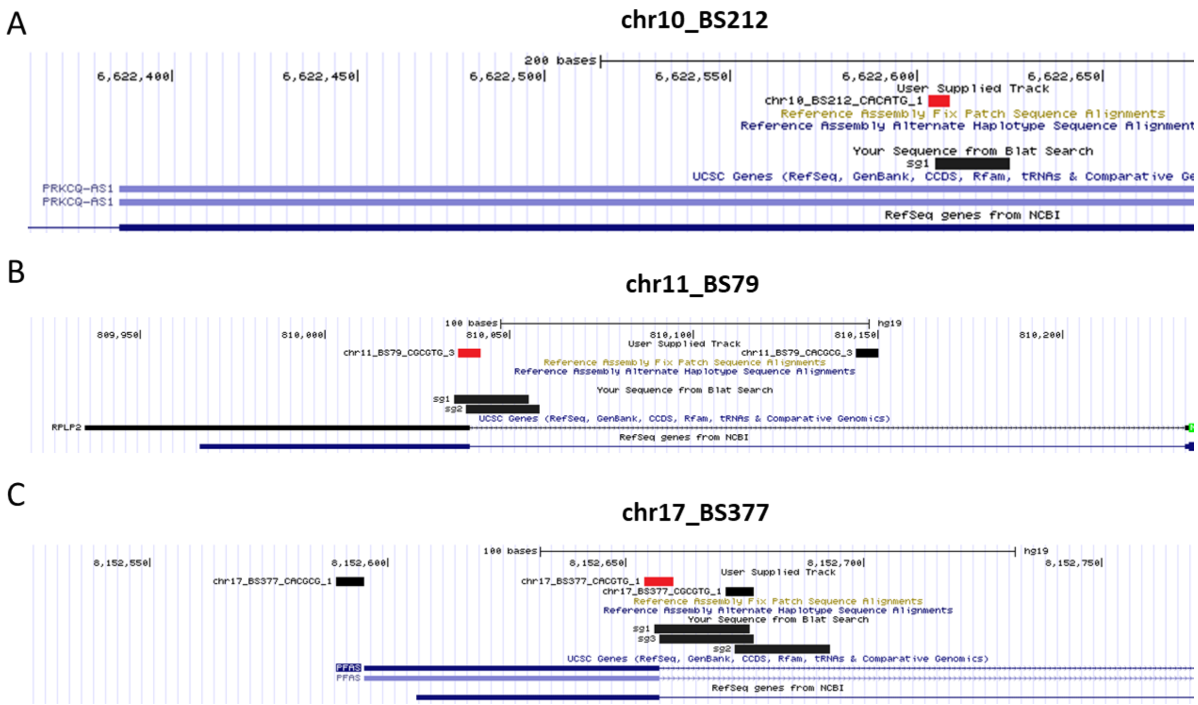

Figure S7. Genomic location of sgRNAs targeting selected E-boxes based on UCSC hg19. Essential E-boxes marked in red.

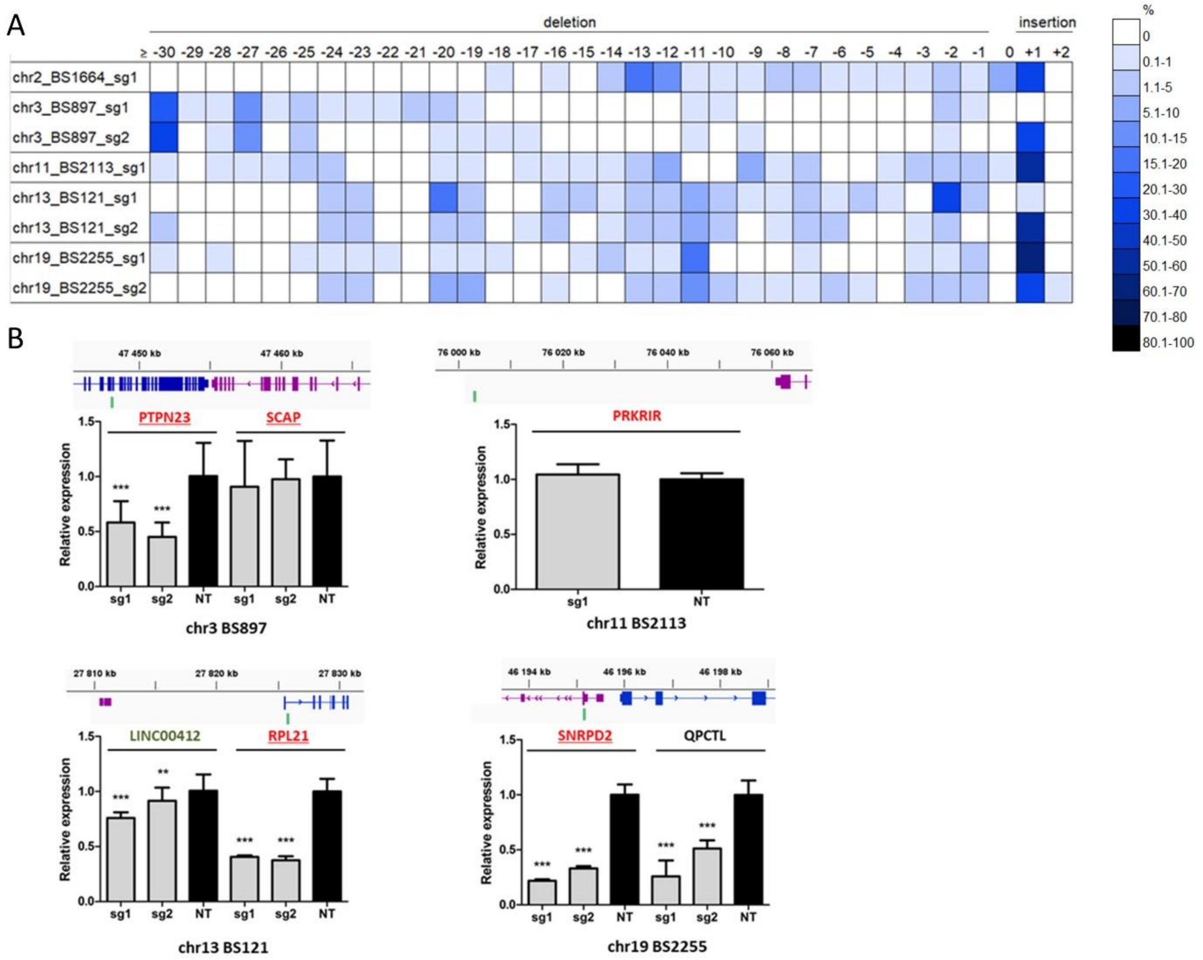

Figure S8. Validation of selected E-boxes in K562 cells. A) Efficiency of disruption of selected E-boxes and the spectrum of mutations introduced by individual sgRNAs, demonstrated by TIDE analysis. Size distribution of introduced indels ranged from ≤-30 bp to +2 bp. Colors indicate percentage of sequences with a given indel size. **B)** qRT-PCR analysis of genes adjacent to selected E-boxes upon CRISPR/Cas9 disruption of E-box sequences. Known MYC-regulated genes are underlined; genes essential or at least 4-fold depleted in Brunello screen are in red. Noncoding genes are in green. NT – average of two non-targeting (negative control) sgRNAs. Mean and SD of two independent experiments, each performed in triplicate, are shown. **, p<0.01; ***, p<0.001, Student's t-test.

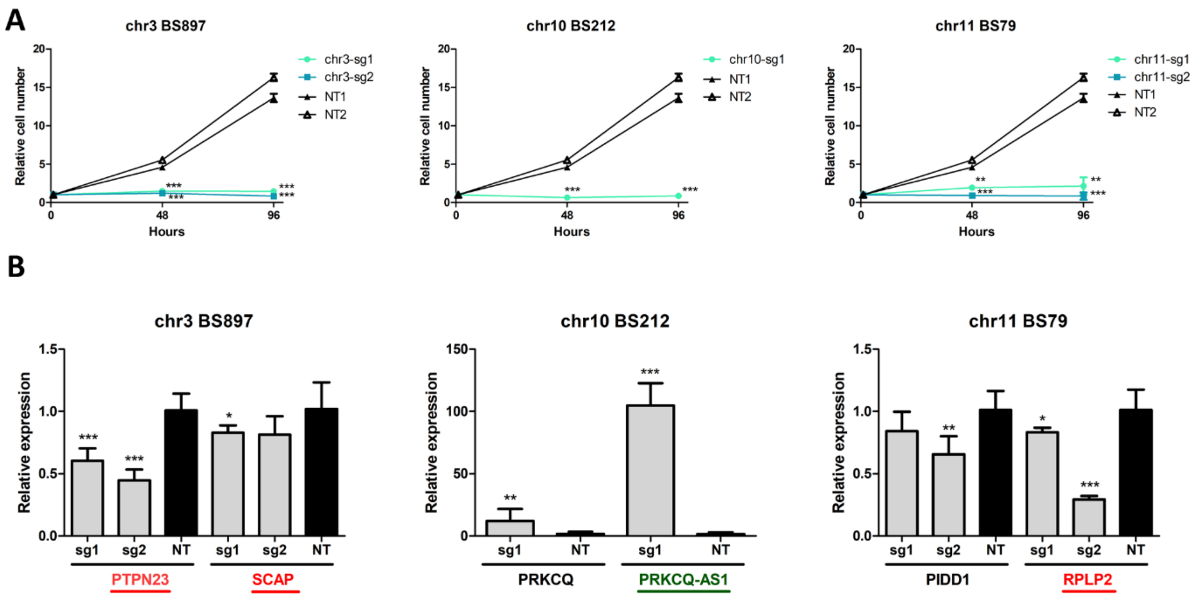

Figure S9. Validation of selected E-boxes in ST486 cells. A) Cell viability upon disruption of selected E-boxes and knockout of adjacent genes was measured using CellTiter-Glo assay at three timepoints: 0, 48 and 96 hours. Shown are mean values and SD from two independent experiments, each performed in triplicate. **, p<0.01; ***, p<0.001, Student's t-test. **B)** qRT-PCR analysis of genes adjacent to selected E-boxes upon CRISPR/Cas9 disruption of E-box sequences. Known MYC-regulated genes are underlined; genes essential or at least 4-fold depleted in Brunello screen are in red. Noncoding genes are in green. NT – average of two non-targeting (negative control) sgRNAs. Mean and SD of two independent experiments, each performed in triplicate, are shown. *, p<0.05; **, p<0.01; ***, p<0.001, Student's t-test.

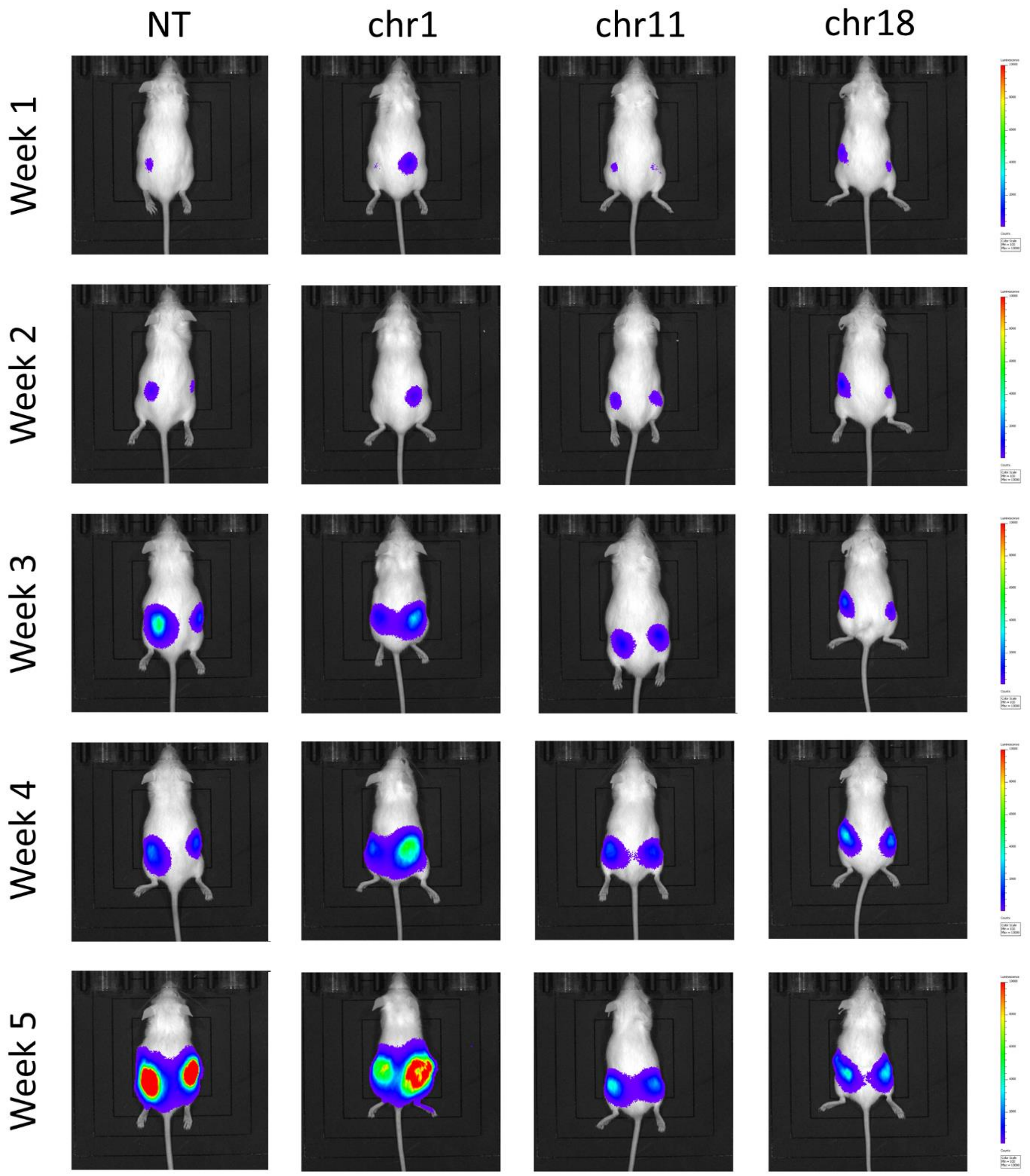

Figure S10. Dynamics of tumor growth in vivo. Representative images of luciferase-based bioluminescence imaging in mouse xenografts of HepG2 cells transduced with sgRNAs targeting E-boxes on chr1, chr11 and chr18, and control (non-targeting) sgRNA. Images were acquired every week for 5 weeks.

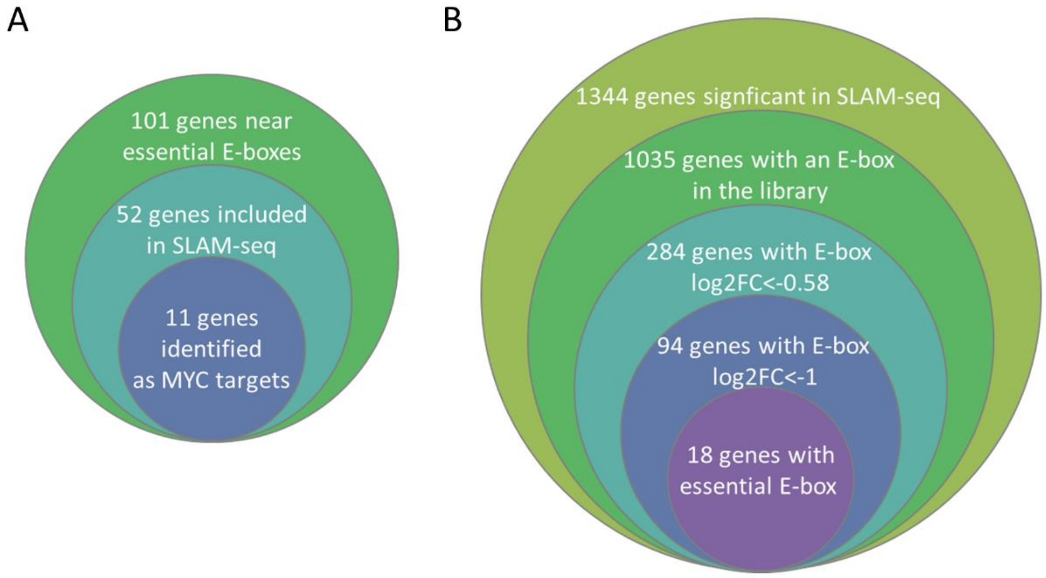

Figure S11. **Intersection of the MYC-CRISPR screen with SLAM-seq data** [1]**. A)** From the viewpoint of 101 genes near essential E-boxes identified in the MYC-CRISPR screen. **B)** From the viewpoint of 1344 genes identified as MYC targets in SLAM-seq.

References

[1] M. Muhar *et al.*, "SLAM-seq defines direct gene-regulatory functions of the BRD4- MYC axis," *Science (80-.).*, vol. 360, no. 6390, pp. 800–805, 2018, doi: 10.1126/science.aao2793.SLAM-seq.
